# Activation of the IKK2/NF-**κ**B pathway in VSMCs inhibits calcified vascular stiffness in CKD

**DOI:** 10.1172/jci.insight.174977

**Published:** 2024-03-12

**Authors:** Shinobu Miyazaki-Anzai, Masashi Masuda, Audrey L. Keenan, Yuji Shiozaki, Jose G. Miranda, Makoto Miyazaki

**Affiliations:** Division of Renal Diseases and Hypertension, Department of Medicine, University of Colorado Anschutz Medical Campus, Aurora, Colorado, USA.

**Keywords:** Vascular biology, Cardiovascular disease, Chronic kidney disease, NF-kappaB

## Abstract

IKK2/NF-κB pathway–mediated inflammation in vascular smooth muscle cells (VSMCs) has been proposed to be an etiologic factor in medial calcification and stiffness. However, the role of the IKK2/NF-κB pathway in medial calcification remains to be elucidated. In this study, we found that chronic kidney disease (CKD) induces inflammatory pathways through the local activation of the IKK2/NF-κB pathway in VMSCs associated with calcified vascular stiffness. Despite reducing the expression of inflammatory mediators, complete inhibition of the IKK2/NF-κB pathway in vitro and in vivo unexpectedly exacerbated vascular mineralization and stiffness. In contrast, activation of NF-κB by SMC-specific IκBα deficiency attenuated calcified vascular stiffness in CKD. Inhibition of the IKK2/NF-κB pathway induced cell death of VSMCs by reducing anti–cell death gene expression, whereas activation of NF-κB reduced CKD-dependent vascular cell death. In addition, increased calcification of extracellular vesicles through the inhibition of the IKK2/NF-κB pathway induced mineralization of VSMCs, which was significantly reduced by blocking cell death in vitro and in vivo. This study reveals that activation of the IKK2/NF-κB pathway in VSMCs plays a protective role in CKD-dependent calcified vascular stiffness by reducing the release of apoptotic calcifying extracellular vesicles.

## Abstract 2

Activation of the IKK2/NFkB cell death pathway in vascular smooth muscle cells modulates calcified vascular stiffness in chronic kidney disease.

## Graphical abstract

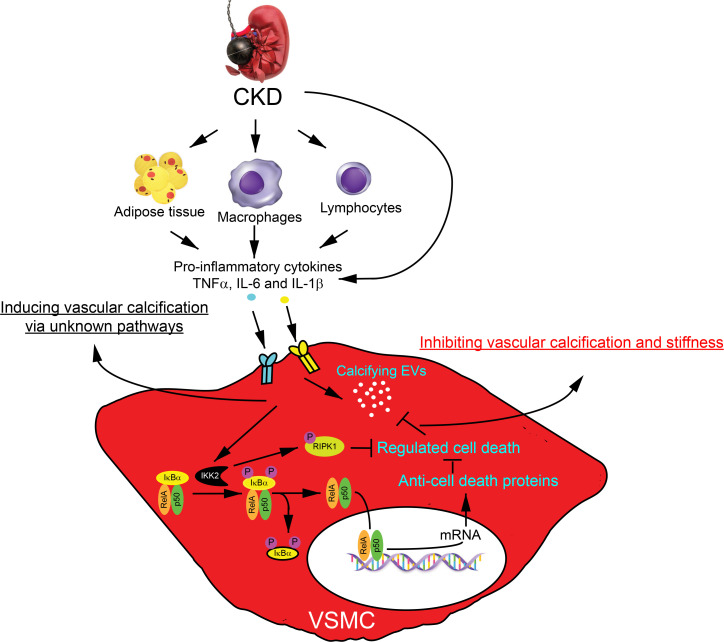

## Introduction

Over half of all deaths among chronic kidney disease (CKD) patients are due to cardiovascular disease (CVD). The risk of CVD mortality in CKD patients is 20–30 times higher than that of the general population (1–4). Growing evidence suggests that this increased risk of CVD mortality is explained by the predisposition of CKD patients to vascular calcification (5–8). Vascular calcification occurs at 2 distinct sites within the vessel wall: the intima and the media. Intimal calcification, also called atherosclerotic calcification, occurs in the context of atherosclerosis and involves lipids, macrophages, and vascular smooth muscle cells (VSMCs) (9, 10). Medial calcification can exist independently of atherosclerosis and is associated with elastin and VSMCs, and is more prevalent in patients with CKD. In both cases, accumulation of calcium phosphate complexes in the vascular wall decreases aortic elasticity and flexibility, which impairs cardiovascular hemodynamics, resulting in substantial morbidity and mortality (7, 11). CKD is represented by states of low-grade chronic inflammation characterized by increased levels of inflammatory markers such as tumor necrosis factor-α (TNF-α) and interleukins (ILs) (7, 12). A number of previous studies show that inflammatory cytokines play causative roles in vascular calcification. Treatment with antiinflammatory agents such anti–TNF-α and anti–IL-1β and –IL-6 monoclonal antibodies have been shown to reduce vascular calcification (13–16). However, the molecular mechanisms by which CKD increases levels of inflammatory factors such as TNF-α and ILs locally in VSMCs, resulting in vascular calcification, have not been fully investigated.

NF-κB proteins comprise a family of structurally related eukaryotic transcription factors that are involved in the control of a large number of normal cellular and organismal processes, such as immune and inflammatory responses (17–19). These transcription factors are highly active in a number of disease states, including cancer, metabolic diseases (e.g., diabetes and obesity), asthma, neurodegenerative diseases, and CVDs (18, 20–24). In most cells, NF-κB is present as a latent, inactive form bound to nuclear factor of κ light polypeptide gene enhancer in B cells inhibitor α (IκBα) in a complex in the cytosol. When a cell receives any of a multitude of extracellular signals such as LPS and TNF-α, NF-κB rapidly enters the nucleus and activates gene expression. Therefore, a crucial step for regulating NF-κB activity is the regulation of the IκBα–NF-κB interaction. Signals that activate NF-κB converge on the activation of a regulatory complex that contains a serine-specific IκB kinase (IKK). IKK is an unusual kinase that contains 3 distinct subunits: IKK1, IKK2, and IKK3. IKK1 and IKK2 are catalytic kinase subunits, while IKK3 is a regulatory subunit that serves as a sensing scaffold and integrator of upstream signals for activation of the catalytic subunits. In the canonical pathway, activation of the IKK complex leads to phosphorylation by IKK2 of 2 specific serines near the N-terminus of IκBα, which targets IκBα for ubiquitination and degradation by the 26S proteasome. The released NF-κB complex can then enter the nucleus to activate the expression of target inflammatory cytokines and chemokine genes (18, 19). Several in vitro and in vivo studies have proposed that NF-κB activation contributes to the etiology of vascular calcification (25–30). However, the VSMC-specific role in the IKK2/NF-κB inflammatory cascade in the regulation of CKD-induced medial calcification has not been fully elucidated.

In addition to inflammation, the IKK2/NF-κB pathway governs cell survival by inhibiting apoptosis, which is a major form of programmed cell death involving multiple caspase reactions (31–34). NF-κB exerts prosurvival effects by inducing the transcription of several antiapoptotic genes such as cIAPs, XIAP, cFLIP, and Bcl2 family members (32, 35, 36). In addition, IKK2 directly inhibits apoptosis by phosphorylating the major proapoptotic factor BAD. Previous studies have shown that apoptosis is an early important event in vascular calcification (37–42). Apoptotic VSMCs disassemble and generate membrane-bound extracellular vesicles (EVs) called apoptotic bodies (ApoBDs, generally 1–5 μm in diameter) (40, 43). The formation of ApoBDs has been proposed to play a critical role in vascular mineralization. In addition, recent studies revealed that apoptosis induces the release of smaller EVs, such as apoptotic microvesicles (0.5–0.2 mm) and exosomes (0.2–0.1 mm) (44–46). In addition, recent evidence on the mechanisms of vascular calcification identified calcifying EV–containing mineralization inducers such as alkaline phosphatase (ALP) derived from VSMCs as the mediators of cardiovascular mineralization (47–50). In this study, we investigated whether CKD induces IKK2/NF-κB–mediated inflammation locally in VSMCs. We examined how the IKK2/NF-κB pathway is involved in the mineralization of VMSCs in vitro using the CRISPR/Cas9 system. We also explored whether changes in the IKK2/NF-κB pathway affect vascular calcification in CKD mouse models.

## Results

**Figure 1:**
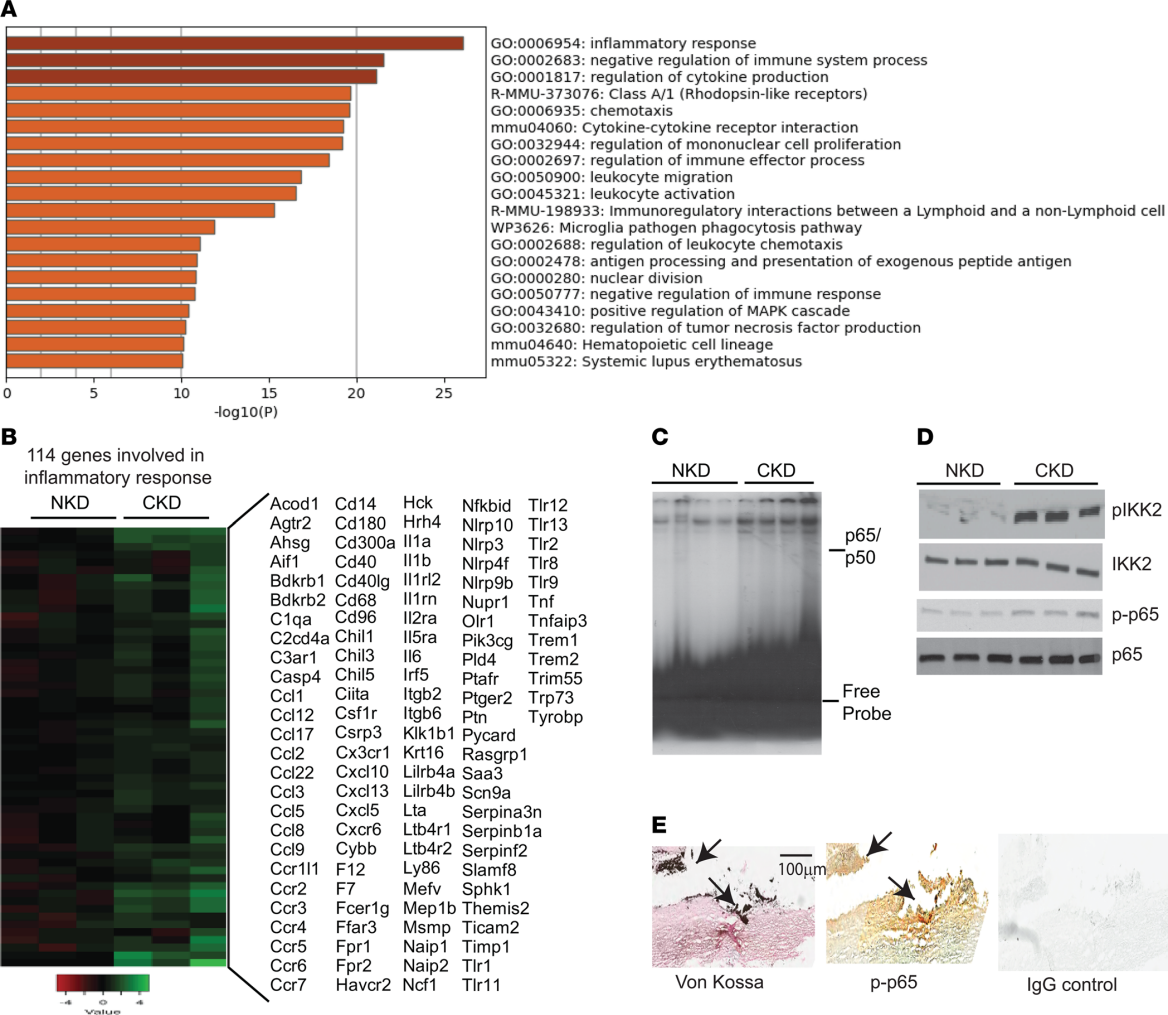
IKK2/NF-κB–mediated proinflammatory pathways were induced in VSMCs by CKD. (**A**) Pathway analysis of mRNA-seq. Eight-week-old male DBA SMMHC-GFP mice were subjected to either 5/6 nephrectomy (CKD) or sham operation (NKD). Aortas were dissected from CKD and NKD mice 12 weeks after the surgeries. SMMHC-GFP^+^ VSMCs were isolated through cell sorting after the digestion of aortas with a collagenase/elastase mixture. Total RNAs were isolated and subjected to mRNA-seq. The mRNA-seq data were deposited to GEO GSE229679. The genes upregulated by CKD were subjected to Metascape pathway analysis (https://metascape.org). (**B**) One hundred fourteen genes involved in the inflammatory response were most strongly induced in the VSMCs of CKD mice. (**C**) Active NF-κB p65-p50 complex was increased in the VSMCs from CKD mice. Levels of p65-p50 complex were analyzed by EMSA. (**D**) Immunoblot analysis of the IKK2/NF-κB pathway. Total protein lysates were isolated from aortic media. (**E**) The serial sections of calcified aorta from a patient with CKD were stained with von Kossa (black = calcified lesion), p-p65 (brown), or IgG (negative control) coupled with an Avidin-Biotin Complex kit (Vector Laboratories). Scale bar: 100 μm.

CKD is represented by states of low-grade chronic inflammation characterized by increased systemic levels of inflammatory markers such as TNF-α and ILs in addition to other complications such as hyperphosphatemia (51, 52). Numerous studies have indicated that local IKK2/NF-κB–mediated inflammation in VSMCs plays a causative role in regulating vascular calcification (25–30). Consistent with our previous studies (14, 38, 39, 42, 53, 54), 5/6-nephrectomized DBA SMMHC-GFP mice had significantly higher levels of serum creatinine compared with sham-operated mice (mice with normal kidney function, NKD), which indicates that 5/6 nephrectomy induces CKD. CKD, but not NKD SMMHC-GFP, mice developed medial calcification (Supplemental Figure 1, A–C; supplemental material available online with this article; https://doi.org/10.1172/jci.insight.174977DS1). In addition, levels of aortic calcium were more than 5-fold higher in CKD mice (Supplemental Figure 1D). To determine whether CKD induced vascular stiffness along with vascular calcification, aortic pulse wave velocity (aPWV) was analyzed with an Indus Doppler Flow Velocity System. The aPWV was greater in CKD compared with NKD mice (Supplemental Figure 1, E and F). These results indicate that CKD induces calcified artery stiffening. To examine whether CKD induces inflammatory signals locally in VSMCs, we performed mRNA-seq using cell sorting on VSMCs from the aortas of CKD and NKD mice. As shown in Figure 1A, the mRNA-seq and pathway analyses confirmed that genes in numerous inflammatory pathways were increased in the VSMCs of CKD mice compared with NKD mice. At the top of the list, levels of 114 genes involved in the inflammatory response were significantly increased in the VSMCs isolated from CKD mice (Figure 1B). Electrophoretic mobility shift assay (EMSA) analysis showed that the NF-κB pathway was drastically activated in the VSMCs of CKD mice. In addition, levels of active phosphorylated IKK2 (p-IKK2) and p65 (p-p65) as well as inflammatory markers (*Il1b*, *Il6*, *Tnfa*, and *iNOS*) were higher in the aortic media of CKD mice (Figure 1, C and D, and Supplemental Figure 1G). To examine whether the NF-κB pathway is activated in the calcified aorta, serial sections of a calcified aorta from a patient with CKD were obtained from the Cooperative Human Tissue Network. As shown in Figure 1, aortic calcified lesions expressed more p-p65 than non-calcified lesions. These data suggest that CKD induces IKK2/NF-κB–mediated inflammation in VSMCs.

**Figure 2:**
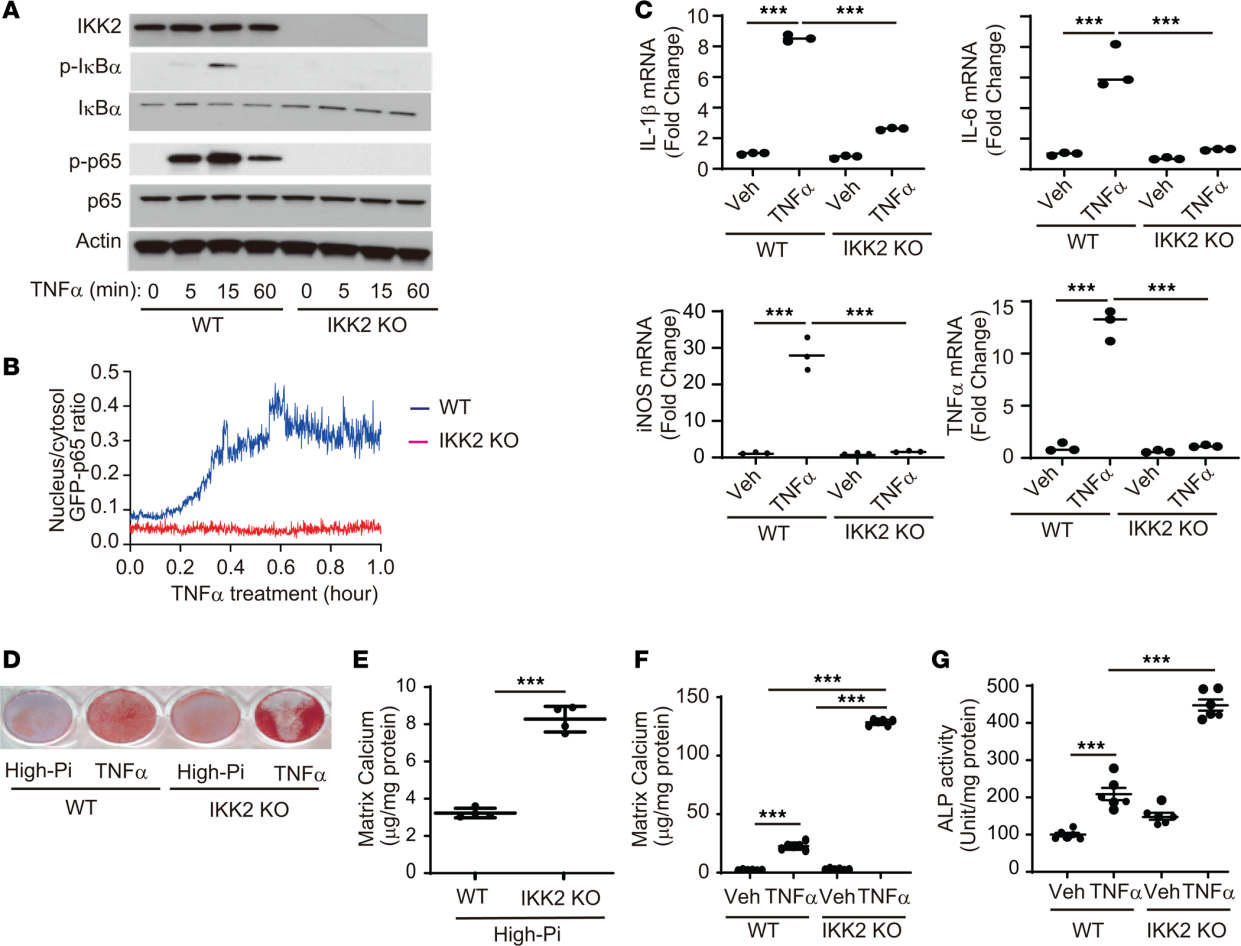
Deletion of the *IKK2* gene induces mineralization of mouse VSMCs despite the inhibition of NF-κB–mediated inflammatory cytokine induction. (**A**) Immunoblot analysis of the IKK2/NF-κB pathway in IKK2-KO VSMCs treated with TNF-α. (**B**) Nuclear p65 translocation was analyzed by transfecting IKK2-KO VMSCs with GFP-p65. The live images are shown in Supplemental Videos 1 and 2. (**C**) mRNA levels of inflammatory markers (IL-1β, IL-6, iNOS, and TNF-α) in IKK2-KO VSMCs treated with vehicle (Veh) or TNF-α. VSMCs were treated with TNF-α in the presence of high phosphate (2.4 mM) for 8 hours. Levels of *36B4* mRNA were used as a control. (**D**–**G**) Alizarin red staining (**D**), levels of matrix calcium (**E** and **F**), and ALP activity (**G**) of VSMCs treated with either high phosphate or TNF-α. VSMCs were treated with either high phosphate (2.4 mM) or TNF-α plus high phosphate for 6 days. ***P < 0.001 by 1-way ANOVA with Tukey’s post hoc test.

CKD induces IKK2/NFκB–mediated inflammation locally in VSMCs. Previous in vitro studies conducted to study the role of IKK2/NF-κB by partial inhibition used chemical inhibitors, dominant negatives, or RNAi (26, 28, 30, 55, 56). To reduce complications from remaining IKK2 activity and off-target effects, we generated IKK2-KO VSMCs using a CRISPR/Cas9 system (Figure 2A). TNF-α treatment induced IKK2-mediated phosphorylation of IκBα and p65 in WT mouse VSMCs, whereas IKK2 deficiency blocked TNF-α–induced phosphorylation of IκBα and p65 (Figure 2A). To confirm whether IKK2 KO blocks the translocation of p65 into the nucleus, VSMCs were transfected with GFP-p65 and live imaging of TNF-α treatment was captured for 1 hour (Supplemental Video 1 for WT VSMCs and Supplemental Video 2 for IKK2-KO VSMCs). IKK2 deficiency completely blocked TNF-α–induced translocation of GFP-p65 into the nucleus (Figure 2B). In addition, IKK2 deficiency abolished inflammatory marker inductions by TNF-α treatment (Figure 2C) in addition to high-phosphate treatment (Supplemental Figure 2, A and B). To mimic CKD conditions in a cell culture system, we treated VSMCs with either high phosphate alone or TNF-α plus high phosphate to analyze the effect of IKK2 deficiency in regulating vascular mineralization and osteogenic differentiation. Unexpectedly, however, IKK2 deficiency aggravated high-phosphate– and TNF-α–induced mineralization (Figure 2, D–F) and osteogenic differentiation of VSMCs (Figure 2G). Similar to CRISPR/Cas9-mediated IKK2 KO, the overexpression of a dominant negative IKK2 kinase-dead (K33M) mutant (*IKK2DN*) in human primary VSMCs led to TNF-α–induced mineralization of human primary VSMCs (Supplemental Figure 2, C and D), suggesting that the anticalcification effect of IKK2 is kinase activity dependent.

**Figure 3:**
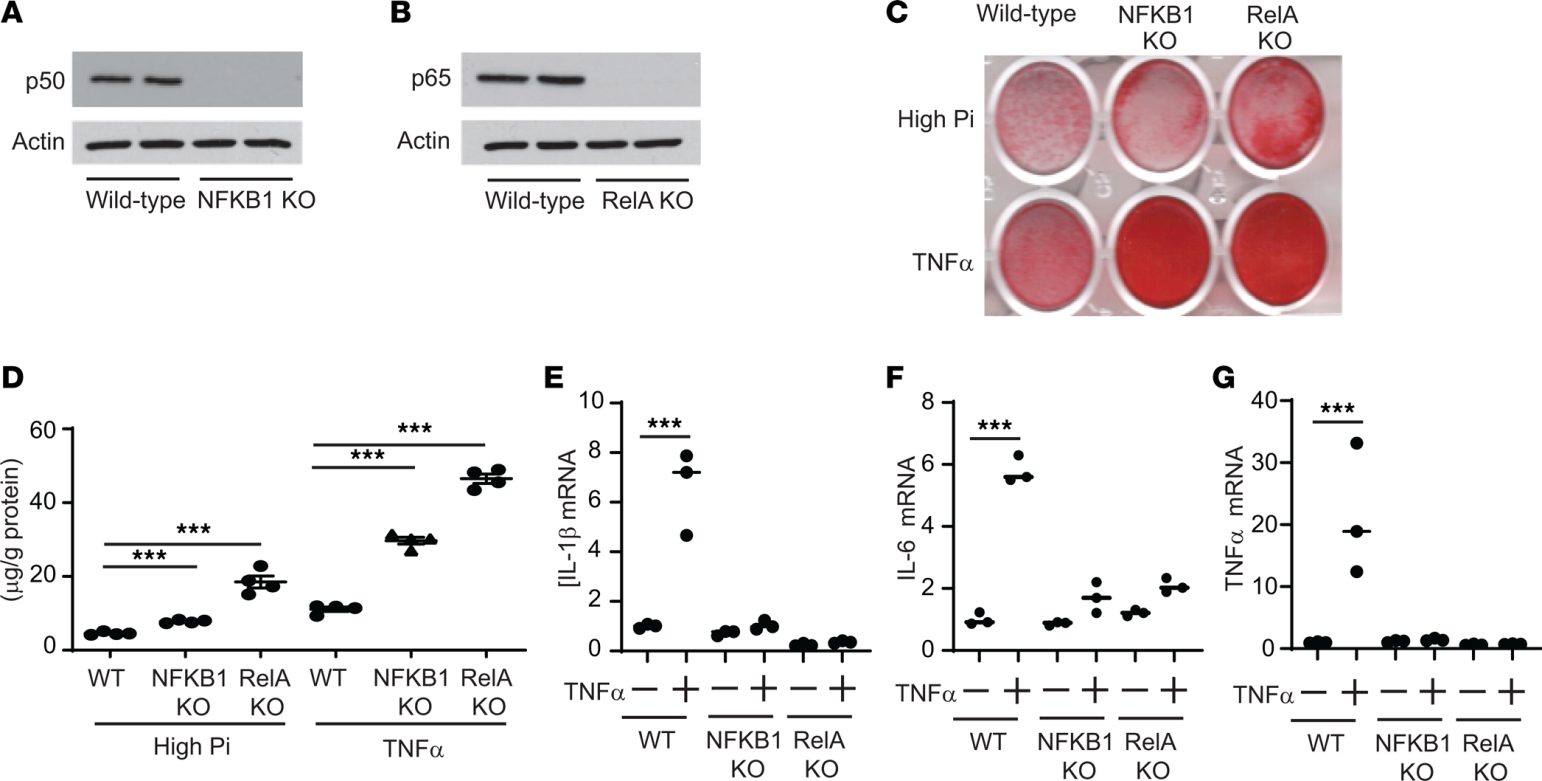
Deletion of NF-κB subunits induces mineralization of VSMCs despite the inhibition of NF-κB–mediated inflammatory cytokine induction. (**A** and **B**) Immunoblot analysis of the p50 and p65 NF-κB subunits in NFKB1-KO and RelA-KO mouse VSMCs, respectively. (**C** and **D**) Alizarin red staining and levels of matrix calcium of VSMCs treated with either high phosphate or TNF-α plus high phosphate. VSMCs were treated with either high phosphate (2.4 mM) or TNF-α plus high phosphate for 6 days. (**E**–**G**) mRNA levels of inflammatory mediators (IL-1β, IL-6, and TNF-α) in IKK2-KO VSMCs treated with TNF-α. VSMCs were treated with TNF-α in the presence of high phosphate (2.4 mM) for 8 hours. Levels of *36B4* mRNA were used as a control. ***P < 0.001 by 1-way ANOVA with Tukey’s post hoc test.

The results from the IKK2-KO VSMCs were unexpected and led us to hypothesize that NF-κB–mediated inflammation has distinct effects on vascular calcification from the IKK2 effect. To address our hypothesis, we next created mouse VSMCs lacking *NFKB1* and *RelA* genes that produce the p50 and p65 NF-κB subunits, respectively (Figure 3, A and B). Similarly to IKK2 deficiency, loss of p50 and p65 NF-κB subunits induced mineralization of mouse VSMCs in response to high-phosphate single and TNF-α plus high-phosphate treatment (Figure 3, C and D). While inducing vascular calcification, p65 deficiency completely reduced levels of inflammatory mediators such as IL-1β, IL-6, and TNF-α (Figure 3, E–G).

**Figure 4:**
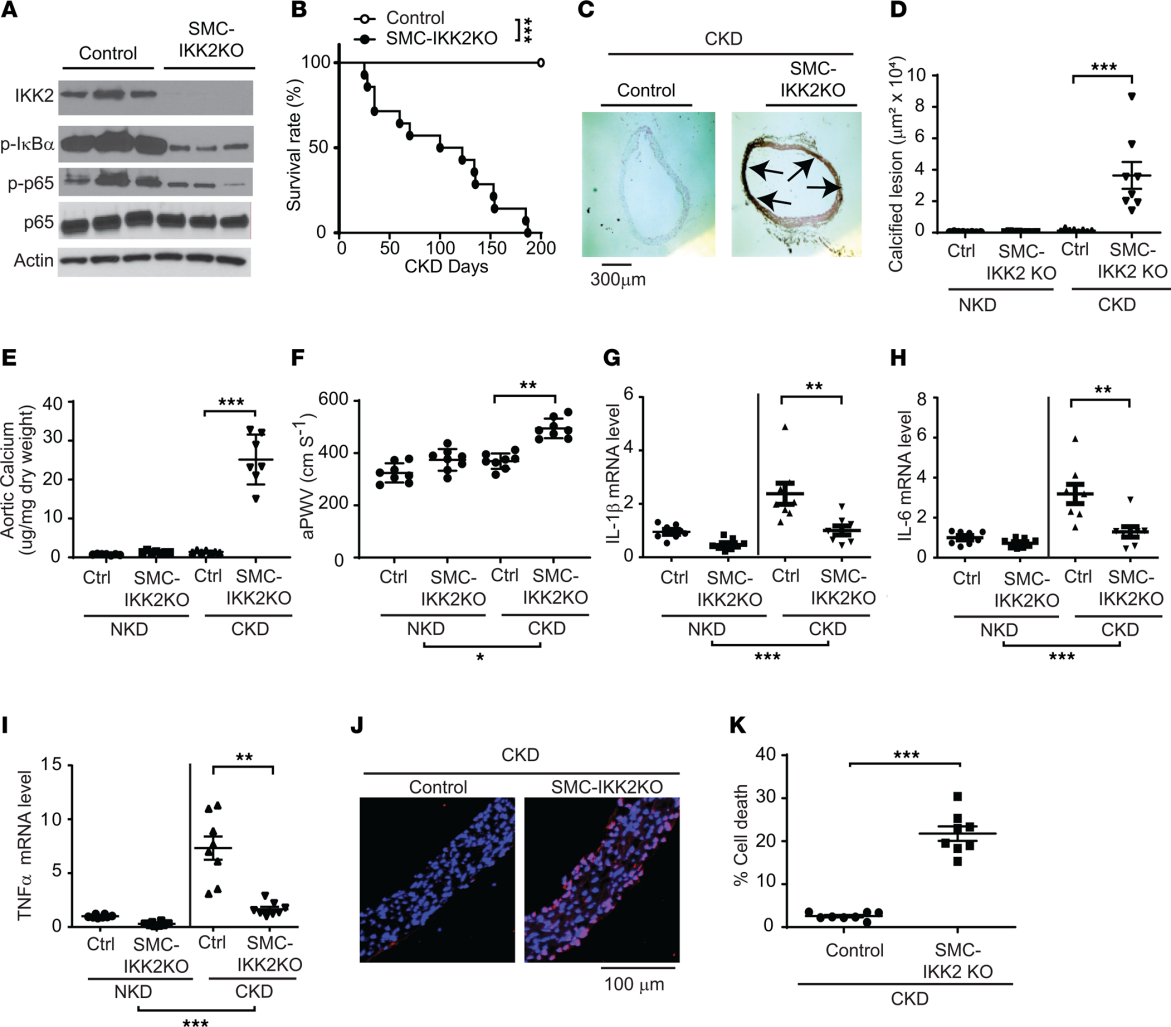
VSMC IKK2 deficiency induces early mortality and calcified vascular stiffness in mice with CKD. (**A**) Immunoblot analysis of the IKK2/NF-κB pathway in the aortic media of SMC-IKK2–KO mice. Eight-week-old control and SMC-IKK2–KO mice (*n* = 8) were intraperitoneally injected with tamoxifen for 5 days and subjected to 5/6 nephrectomy (CKD). Three weeks after the surgeries, animals were euthanized. (**B**) Survival rate of SMC-IKK2–KO mice under CKD. (**C**) Histological and (**D**) quantitative analysis of aortic arches with von Kossa staining. Aortas were dissected from the mice 3 weeks after CKD was induced. Arrows (black) indicate calcified lesions. Scale bar: 300 μm. (**E**) Aortic calcium content. Aortic calcium content was analyzed with an ash assay coupled with a colorimetric calcium assay. (**F**) aPWV was analyzed using an Indus Doppler Flow Velocity System 3 weeks after CKD was induced. (**G**–**I**) mRNA levels of inflammatory markers IL-1β (**G**), IL-6 (**H**), and TNF-α (**I**) in the aortic media of SMC-IKK2–KO mice under NKD and CKD. Aortic media were dissected from the mice 3 weeks after CKD was induced. (**J** and **K**) Aortic cell death. Cell death was analyzed with a Roche in situ cell death kit. Scale bar: 100 μm. **P < 0.01; ***P < 0.001 by log-rank test (**B**) or 1-way ANOVA with Tukey’s post hoc test (**D**–**I** and **K**).

To examine whether modulations of the IKK2/NF-κB pathway in VSMCs in vivo affects CKD-dependent vascular calcification, we generated tamoxifen-inducible VSMC-specific *IKK2*-KO (SMC-IKK2–KO) mice and subjected them to 5/6 nephrectomy to induce CKD. Tamoxifen injection was able to abolish the expression of IKK2 in the aortic media (Figure 4A), but not adventitia or other tissues (Supplemental Figure 3, A and B) of SMC-IKK2–KO mice. The significant reduction in IKK2 expression reduced levels of p-IκBα and p-p65 in the media that mediate the downstream phosphorylation signaling of IKK2 (Figure 4A). Strikingly, CKD significantly induced early mortality of SMC-IKK2–KO mice more than control mice (Figure 4B). Because of the CKD-induced early death of SMC-IKK2–KO mice, we examined whether SMC-IKK2 deficiency accelerates CKD-dependent vascular calcification and stiffness at an early time point (3 weeks after CKD induction) when control mice do not develop CKD-dependent cardiovascular complications (Figure 4, C–F). Serum creatinine was increased 2.3-fold by 5/6 nephrectomy in both control and SMC-IKK2–KO mice, whereas levels of serum triglyceride were significantly lower in both control and SMC-IKK2–KO mice with CKD (Supplemental Table 1). Histological analysis of the aortic arches with von Kossa stain revealed that SMC-IKK2 deficiency severely aggravated medial calcification under CKD. Calcified lesions and aortic calcium content were approximately 150-fold and 20-fold greater in CKD SMC-IKK2–KO mice than CKD control mice (Figure 4, D and E). In addition, the aPWV was greater in CKD SMC-IKK2–KO mice compared with other groups (Figure 4F). qPCR analysis showed that CKD-induced inflammatory mediators such as IL-1β, IL-6, and TNF-α were normalized by SMC-IKK2 deficiency (Figure 4, G–I). We have previously shown that CKD induces vascular cell death. SMC-IKK2 deficiency remarkably induced cell death in the aortic media (Figure 4, J and K).

**Figure 5:**
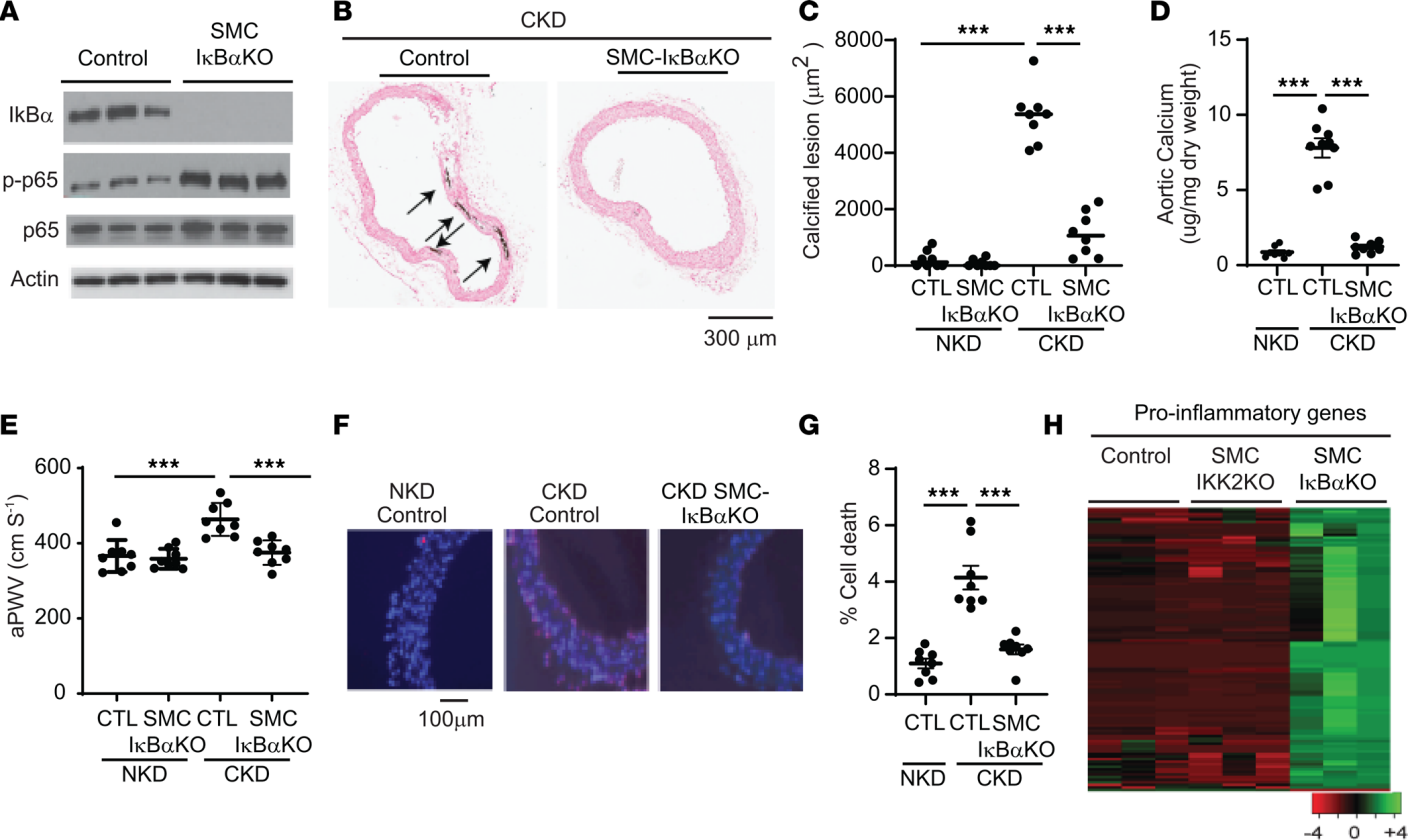
VSMC IκB deficiency attenuates CKD-induced calcified vascular stiffness. (**A**) Immunoblot analysis of the IKK2/NF-κB pathway in the aortic media of SMC-IκBα–KO mice. Eight-week-old control and SMC-IκBα–KO mice (*n* = 8) were intraperitoneally injected with tamoxifen for 5 days and subjected to 5/6 nephrectomy (CKD). Twelve weeks after the surgeries, animals were euthanized. (**B** and **C**) Histological analysis of aortic arches with von Kossa staining. Aortas were dissected from the mice 12 weeks after sham or 5/6 nephrectomy operations. Arrows (black) indicate calcified lesions. Scale bar: 300 μm. (**D**) Aortic calcium content. Aortic calcium content was analyzed with an ash assay coupled with a colorimetric calcium assay. (**E**) aPWV was analyzed using an Indus Doppler Flow Velocity System 12 weeks after CKD was induced. (**F** and **G**) Aortic cell death. Cell death was analyzed with a Roche in situ cell death kit. Aortic media were dissected from the mice 3 weeks after CKD was induced. Pink indicates cell death. Scale bar: 100 μm. (**H**) Heatmap with mRNA levels of more than 100 inflammatory genes induced by CKD in the aortic media of SMC-IKK2–KO and SMC-IκBα–KO mice under CKD. Aortic medial layers were dissected 3 weeks after CKD was induced. The numbers are shown in Supplemental Table 3. ***P < 0.001 by 1-way ANOVA with Tukey’s post hoc test.

Since inhibition of the IKK2/NF-κB pathway unexpectedly worsened CKD-dependent vascular complications in in vitro and in vivo models, we next examined how activation of the NF-κB pathway affects CKD-dependent vascular complications by knocking out IκBα, which is a central inhibitor of the NF-κB pathway. Since global IκBα-KO mice were embryonic lethal, we generated SMC-specific IκBα-KO mice by inserting 2 *loxP* sites into intron 1 and intron 5 and crossing with tamoxifen-inducible SMMHC-creER^T2^ (Supplemental Figure 3C). Similar to SMC-IKK2 deficiency, tamoxifen injections completely removed IκBα from the aortic media of SMC-IκBα–KO mice but not control mice, resulting in a significant increase in an active NF-κB subunit, p-p65 (Figure 5A). Levels of IκBα protein and mRNA in the aortic adventitia, endothelial layer, and peritoneal macrophages were not different between control and SMC-IκBα–KO mice (Supplemental Figure 3, D and E). Unlike SMC-IKK2–KO mice, CKD did not affect the mortality of SMC-IκBα–KO mice (data not shown). The mice were therefore euthanized 12 weeks after CKD was induced, when the control mice develop CKD-dependent vascular complications. CKD increased levels of serum phosphorus and creatinine, while levels of serum triglycerides and calcium were reduced in both control and SMC-IκBα–KO mice (Supplemental Table 2). Under NKD, aortic calcification was comparable between WT and SMC-IκBα–KO mice, whereas SMC-specific IκBα deficiency significantly attenuated vascular calcification, stiffness, and cell death (Figure 5, B–G) under CKD. As shown in Figure 1, CKD induced 116 inflammatory markers. We next examined whether these CKD-induced inflammatory markers were induced by SMC-IκBα deficiency and reduced by SMC-IKK2 deficiency. SMC-IκBα deficiency significantly induced approximately 90% of the CKD-induced inflammatory markers, whereas SMC-IKK2 deficiency reduced approximately 50% of the CKD-induced inflammatory markers (Figure 5H and Supplemental Table 3).

**Figure 6:**
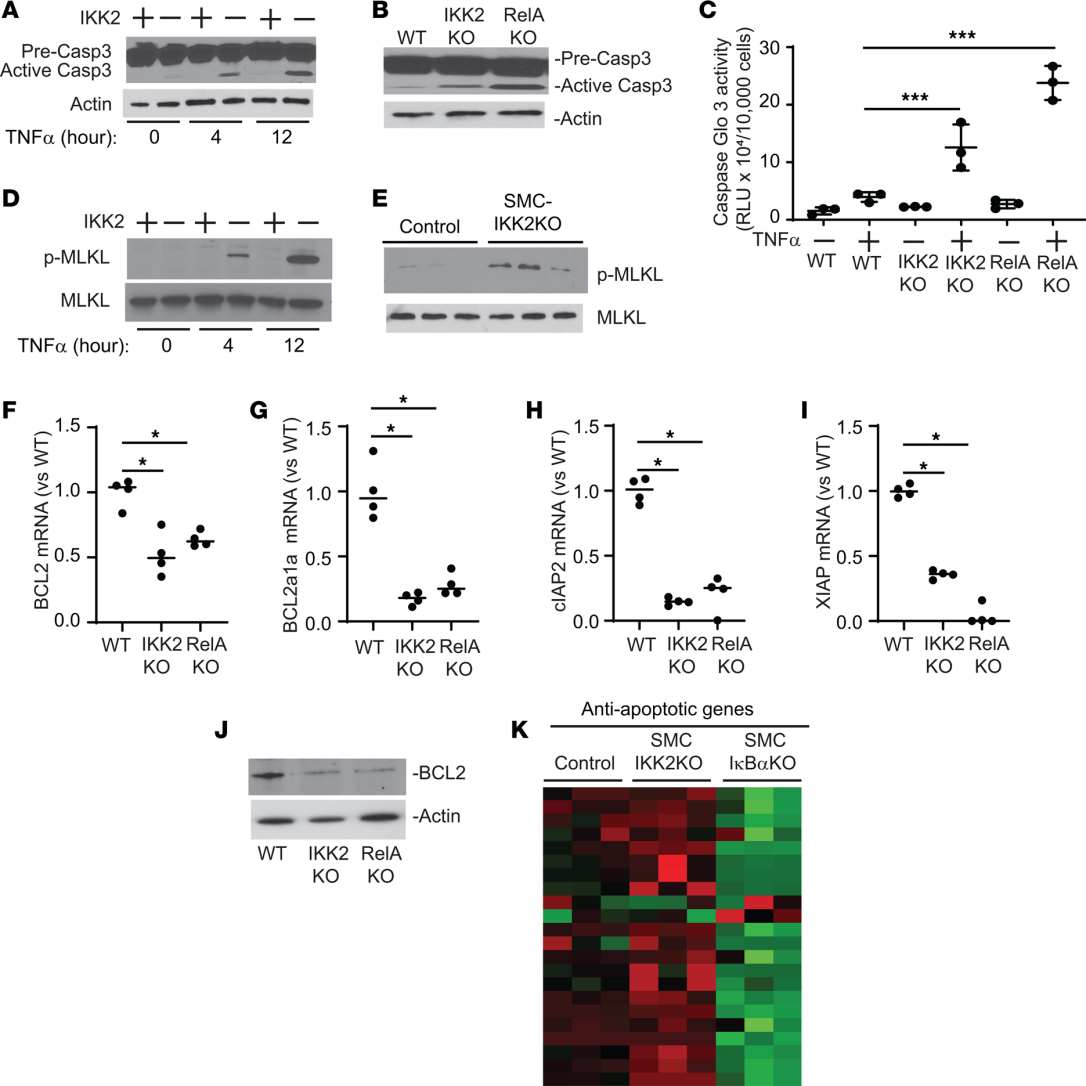
Deletion of the IKK2/NF-κB pathway induces apoptosis of VSMCs. (**A**) Immunoblot analysis of caspase 3 (Casp3) in IKK2-KO VSMCs treated with TNF-α. VMSCs were treated with TNF-α plus high phosphate (2.4 mM) for 6 hours and 12 hours. (**B**) Immunoblot analysis of Casp3 in IKK2-KO and RelA-KO VSMCs treated with high phosphate. VMSCs were treated with high phosphate (2.4 mM) for 12 hours. (**C**) Casp3 activity in IKK2-KO and RelA-KO VSMCs treated with TNF-α. VMSCs were treated with TNF-α plus high phosphate (2.4 mM) for 12 hours. (**D**) Immunoblot analysis of p-MLKL in IKK2-KO VSMCs treated with TNF-α. VMSCs were treated with TNF-α plus high phosphate (2.4 mM) for 6 hours and 12 hours. (**E**) Immunoblot analysis of p-MLKL in the aortic media of CKD SMC-IKK2–KO mice. (**F**–**I**) mRNA levels of antiapoptotic proteins in IKK2-KO and RelA-KO VSMCs. (**J**) Immunoblot analysis of Casp3 in IKK2-KO and RelA-KO VSMCs. VMSCs were treated with TNF-α plus high phosphate (2.4 mM) for 12 hours. (**K**) Heatmap with mRNA levels of 21 antiapoptotic proteins in the aortic media of SMC-IKK2–KO and SMC-IκBα–KO mice. Aortic medial layers were dissected 3 weeks after CKD was induced. The numbers are shown in Supplemental Table 4. *P < 0.05; ***P < 0.001 by 1-way ANOVA with Tukey’s post hoc test.

**Figure 7:**
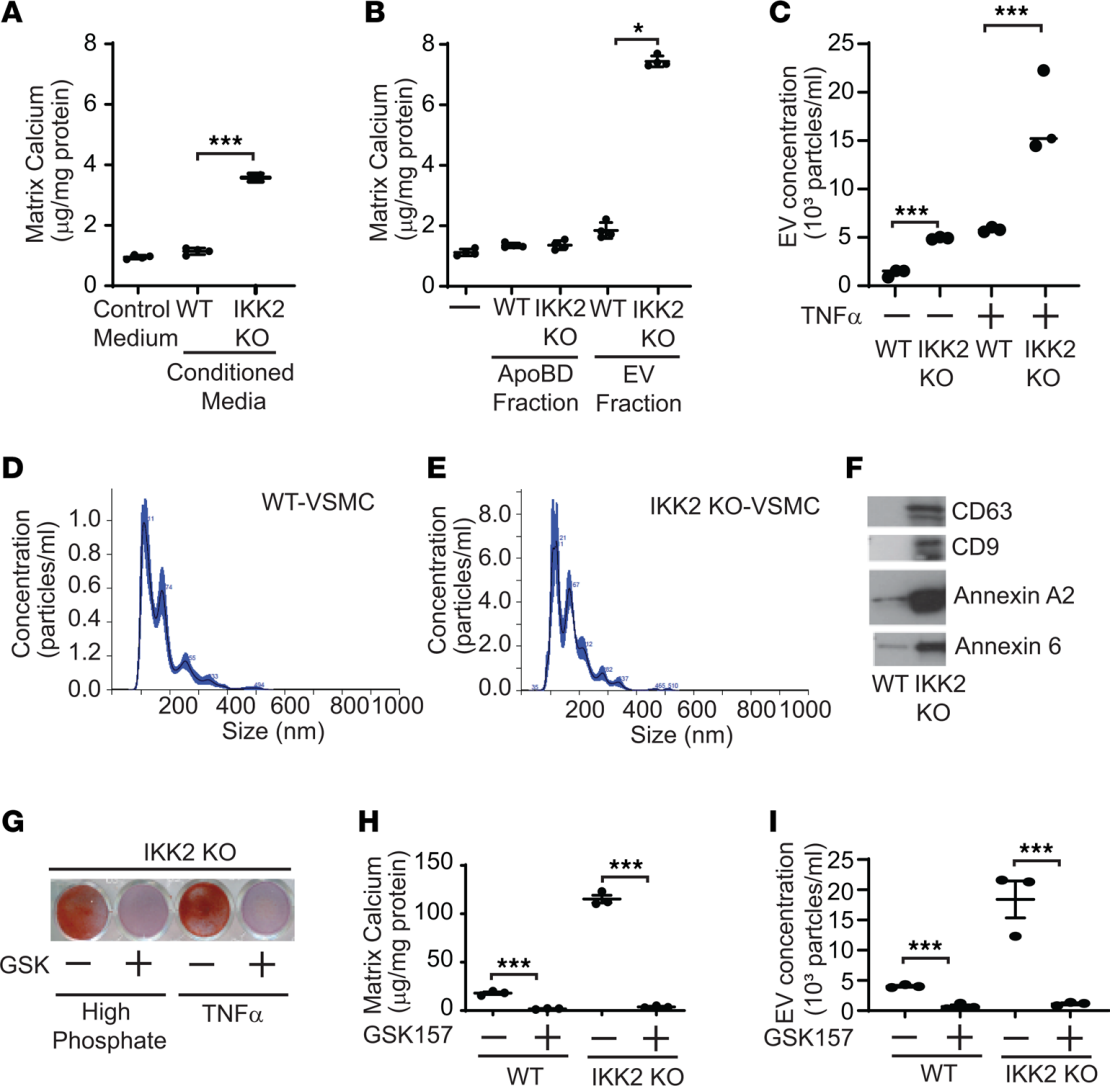
Deletion of IKK2 induces the secretion of calcifying extracellular vesicles, which is completely inhibited by cell death inhibitor. (**A**) Conditioned media from IKK2-KO VSMCs but not WT VSMCs induces mineralization of WT VSMCs. The conditioned media were collected from IKK2-KO and WT VSMCs treated with TNF-α plus high phosphate (2.4 mM) for 48 hours in the presence of EV-depleted FBS. WT VSMCs were treated with the conditioned media for 7 days. (**B**) The EV-enriched fraction, but not the ApoBD-enriched fraction, induces mineralization of VSMCs. WT VSMCs were treated with 40 mg/mL ApoBD- and EV-enriched fractions from IKK2-KO and WT VSMC cultures for 7 days in the presence of 0.5% EV-free FBS. ApoBD-enriched and EV-enriched fractions were isolated with sequential centrifugation. (**C**) EV concentration and (**D** and **E**) size in IKK2-KO VSMCs. EV concentration was determined with NTA using a NanoSight NS500. VSMCs were treated with TNF-α plus high phosphate (2.4 mM) for 48 hours. (**F**) Immunoblot analysis of EV markers (CD63, CD9, annexin A2, and annexin 6) in the culture media of IKK2-KO VSMCs. VSMCs were treated with TNF-α plus high phosphate (2.4 mM) for 48 hours. Culture media (20 mL) were subjected to immunoblot analysis. The densitometry quantifications are shown in Supplemental Figure 4. (**G**) Alizarin red staining, (**H**) matrix calcium content, and (**I**) EV concentration in IKK2-KO VSMCs treated with cell death inhibitor. VSMCs were treated with high phosphate or TNF-α plus high phosphate in the absence and presence of cell death inhibitor (0.1 μM GSK2656157) for 7 days for calcium analysis and 2 days for EV analysis. *P < 0.05; ***P < 0.001 by 1-way ANOVA with Tukey’s post hoc test.

**Figure 8:**
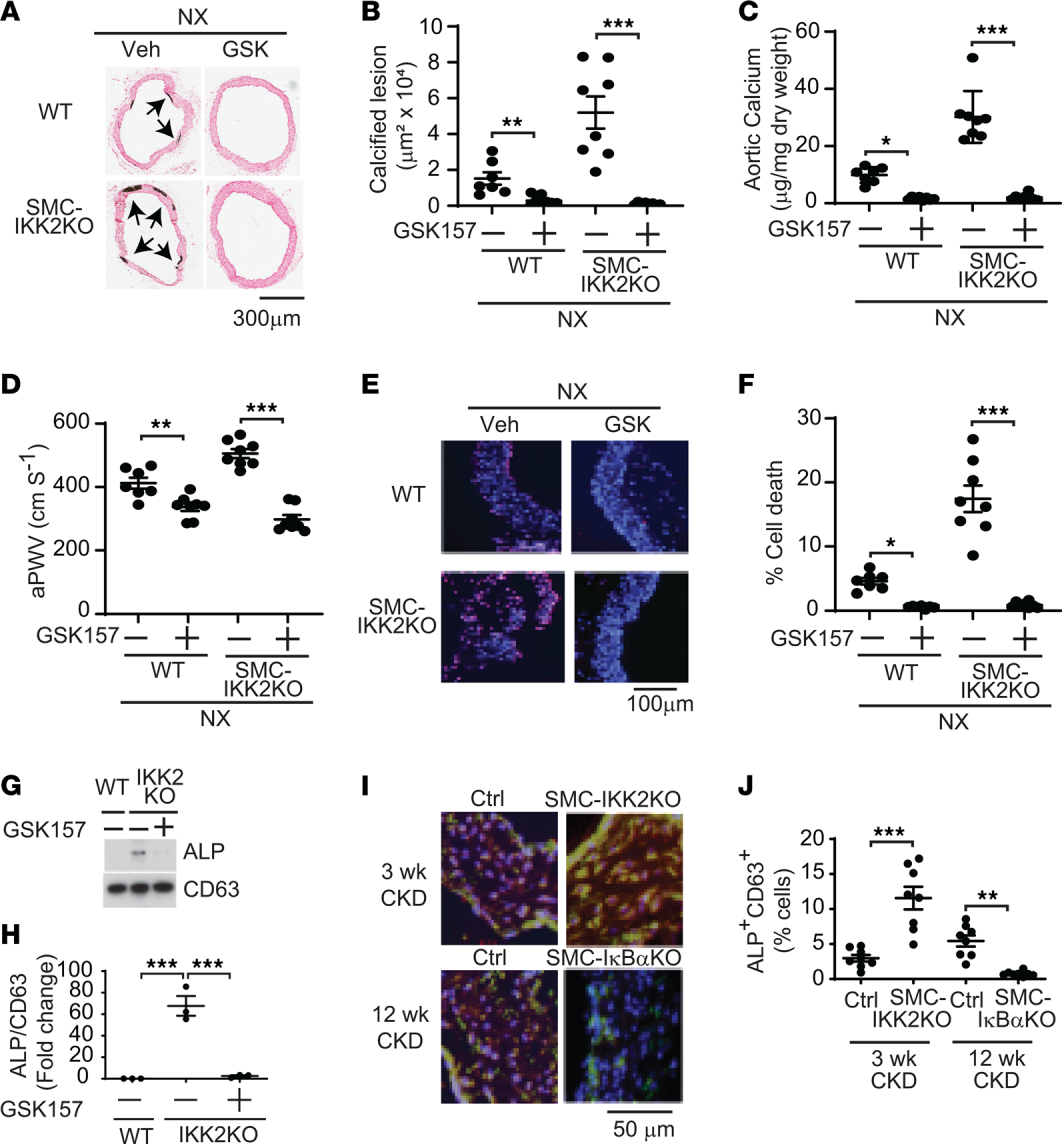
Inhibition of cell death completely attenuates CKD-dependent calcified vascular stiffness. (**A** and **B**) Quantitative histological analysis of aortic arches with von Kossa staining. Aortas were dissected from CKD SMC-IKK2–KO and WT mice (*n* = 7–8) treated daily with 0.5 mg/kg body weight GSK2656157 for 3 weeks and 12 weeks, respectively. Arrows (black) indicate calcified lesions. Scale bar: 100 μm. (**C**) Aortic calcium content. Aortic calcium content was analyzed with an ash assay coupled with a colorimetric calcium assay. (**D**) aPWV was analyzed using an Indus Doppler Flow Velocity System. (**E** and **F**) Aortic cell death. Cell death (pink; nuclei stained blue) was analyzed with a Roche in situ cell death kit. Scale bar: 100 μm. (**G**) Immunoblot analysis and (**H**) quantification of ALP in the EV fraction of IKK2-KO VSMCs treated with cell death inhibitor for 48 hours. (**I** and **J**) Immunofluorescence analysis of aortic calcifying EVs in SMC-IKK2–KO and SMC-IκBα–KO mice. CD63 (green) and ALP (red) double-positive areas (yellow, CD63^+^ALP^+^) were analyzed as calcifying EVs. Scale bar: 50 μm. *P < 0.05; **P < 0.01; ***P < 0.001 by 2-tailed Student’s *t* test (**B**, **C**, **D**, **F**, and **J**) or 1-way ANOVA with Tukey’s post hoc test (**H**).

Severe vascular cell death was associated with CKD-induced and SMC-IKK2 deficiency–induced vascular calcification. The IKK2/NF-κB pathway is a critical inhibitory regulator of apoptotic cell death by inducing antiapoptotic genes in addition to inflammation (31, 32). We next examined whether the inhibition of the IKK2/NF-κB pathway induces apoptotic cell death in VSMCs. IKK2 and RelA deficiency enhanced TNF-α–induced apoptosis (Figure 6, A–C). TNF-α treatment time-dependently activated caspase 3 (Casp3) in IKK2-KO VSMCs more than WT VSMCs (Figure 6A). RelA deficiency also enhanced the activation of Casp3 (Figure 6B). Upon TNF-α treatment, both IKK2-KO and RelA-KO VSMCs had significantly higher Casp3 activity (Figure 6C). In addition to increased active Casp3, IKK2 deficiency increased levels of the active terminal enzyme of necroptosis, phosphorylated MLKL, in VSMCs treated with TNF-α (Figure 6D) and in the aortic media (Figure 6E). These data suggest that CKD and IKK2 deficiency simultaneously induce apoptosis and necroptosis in VSMCs. VSMCs lacking IKK2 and RelA genes had significantly lower levels of several antiapoptotic genes and proteins such as *Bcl2*, *Bcl2a1a*, *cIAP2*, and *XIAP* (Figure 6, F–J). Consistently, under CKD, SMC-IκBα deficiency induced 21 out of 22 antiapoptotic genes expressed in VSMCs, whereas SMC-IKK2 deficiency significantly reduced 18 antiapoptotic genes (Figure 6K and Supplemental Table 4). Recent studies indicated that apoptosis increases the secretion of EVs such as ApoBD and apoptotic EVs (44–46). In addition, there is growing evidence that the formation and secretion of calcifying vesicles are critical steps for vascular mineralization (47–50). We next examined whether IKK2 deficiency induces the secretion of calcifying mediators such as ApoBD. Treatment of WT VSMCs with 48-hour-conditioned media from IKK2-KO VSMC cultures significantly induced levels of matrix calcium contents by 3.8-fold compared with treatment with media from WT VSMC cultures (Figure 7A), suggesting that IKK2 deficiency induces the secretion of calcifying factors. Since ApoBD is known to induce mineralization of VSMCs (40), the conditioned media from WT and IKK2-KO VSMCs was fractionated by ultracentrifuge. Interestingly, treatment of WT VSMCs with ApoBD-enriched fractions from either WT or IKK2-KO cultures did not affect mineralization of VSMCs, whereas EV-enriched fractions from IKK2-KO VSMC cultures, but not WT VSMC cultures, significantly induced the mineralization of VSMCs (Figure 7B). Nanoparticle tracking analysis (NTA) revealed that IKK2 deficiency enhanced the secretion of EVs by approximately 5-fold under normal and TNF-α–treated conditions (Figure 7C). IKK2 deficiency did not affect the size distribution of EVs compared with WT VSMCs (Figure 7, D and E). To confirm that IKK2 deficiency increases the secretion of EVs, levels of EV markers in the culture media were determined by immunoblot analysis. Consistent with NTA, IKK2 deficiency increased levels of CD63, CD9, annexin A2, and annexin 6 in the culture media by 185-fold, 697-fold, 57-fold, and 12-fold, respectively (Figure 7F and Supplemental Figure 4, A–D). We next examined whether cell death is linked to IKK2-KO–mediated mineralization, osteogenic differentiation, and EV secretion. Recent studies demonstrated that treatment with the cell death inhibitor GSK2656157 (GSK157) completely blocked TNF-α–induced cell death. As shown in Figure 7, G and H, and Supplemental Figure 4, E and F, treatment with GSK157 totally blocked TNF-α– and high-phosphate–induced mineralization and osteogenic differentiation of both WT and IKK2-KO mice. Blocking cell death significantly reduced the secretion of EVs from WT and IKK2-KO cells (Figure 7I). We next examined whether treatment of WT and SMC-IKK2–KO mice with GSK157 in vivo blocks calcified vascular stiffness and cell death induced by CKD. As shown in Figure 8, A–F, GSK157 treatment significantly attenuated CKD-dependent vascular calcification, vascular stiffness, and cell death in both WT and SMC-IKK2–KO mice. IKK2 deficiency increased levels of ALP protein in CD63^+^ EVs, but that was significantly reduced by blocking cell death (Figure 8, G and H), suggesting that calcifying EVs were increased by IKK2-KO–mediated cell death. We also tested whether IKK2/NF-κB modulation affects levels of calcifying EVs in the aortic media in vivo. Immunofluorescence analysis of the aortic sinuses showed that the number of CD63^+^ALP^+^ double-positive areas were increased in the aortic media of CKD SMC-IKK2–KO mice and reduced by SMC-IκBα deficiency (Figure 8, I and J).

## Discussion

IKK2/NF-κB signaling is activated by numerous discrete stimuli and is a master regulator of the inflammatory response (17–19). Activation of the IKK2/NF-κB pathway has been proposed to contribute to the etiology of cardiovascular complications in CKD, such as vascular calcification (24–30). However, the VSMC-specific role of IKK2/NF-κB in the regulation of CKD-induced vascular complications is still obscure. In this study, mRNA-seq analysis coupled with cell sorting, immunoblot analysis, and EMSA analysis revealed that CKD induces more than 100 genes involved in the inflammatory response locally in VSMCs via activation of the IKK2/NF-κB pathway. Proinflammatory genes increased by CKD were associated with medial calcification and vascular stiffness. Based on previous studies from other groups, we expected that inhibition of the NF-κB pathway would attenuate vascular mineralization by reducing the production of proinflammatory mediators locally from VSMCs. In fact, all of the in vitro and in vivo models tested in this study suggest that activation of IKK2/NF-κB signaling by CKD induces inflammatory mediator expression in VSMCs. Unexpectedly, however, we demonstrated that activation of the IKK2/NF-κB pathway in VSMCs elicits strong protective effects for CKD-dependent vascular complications. Using the CRISPR/Cas9 technique, we first deleted 3 key molecules (*IKK2*, *RelA*, and *NFKB1*) involved in IKK2/NF-κB signaling from cultured VSMCs. All of the gene deletions aggravated TNF-α– and high-phosphate–induced mineralization and osteogenic differentiation of VSMCs. We used 2 mouse models to modulate the IKK2/NF-κB pathway specifically in VSMCs. SMC-IKK2–KO mice had significantly reduced CKD-induced NF-κB activation and proinflammatory mediator expression in VSMCs, and SMC-specific IKK2 deficiency accelerated CKD-induced medial calcification. On the other hand, SMC-IκBα deficiency attenuated CKD-mediated vascular complications despite the activation of NF-κB and proinflammatory mediator expression in VSMCs. These findings strongly suggest that the activation of IKK2/NF-κB signaling in VSMCs is a defense mechanism against calcified vascular stiffness in CKD.

We provided mechanistic insights into the protective role of IKK2/NF-κB signaling in regulating CKD-mediated vascular calcification. A number of recent studies have proposed that the secretion of calcifying EVs containing CD63, annexins, and ALP is a pivotal event in the pathogenesis of vascular osteogenesis and mineralization (48–50). Our studies reveal that the inhibition of IKK2 increased the secretion of calcifying EVs. Mechanistically, cell death by the inhibition of the IKK2/NF-κB pathway activates the secretion of calcifying EVs from VSMCs, resulting in vascular mineralization and osteogenesis. It was previously considered that the secretion of ApoBD mediates cell death–induced VSMC mineralization. Since ApoBD fractions of IKK2-KO cells did not influence the mineralization of VSMCs, ApoBD plays a minor role in the IKK2/NF-κB cell death/mineralization cascade. In this study, we used GSK157 as a cell death inhibitor because the chemical completely and potently (~nM level) blocks TNF-α–mediated cell death by inhibiting multiple protein kinases such as PERK and RIPK1 (57). We have previously shown that PERK-mediated ER stress is involved in TNF-α–mediated vascular calcification (14). Interestingly, cell death inhibitor treatment blocked not only TNF-α–mediated, but also high-phosphate–mediated VSMC mineralization. GSK157 also inhibited other uremic toxin–induced (cresol sulfate and indoxyl sulfate) mineralization. More importantly, RIKP1 inhibitor completely blocked CKD-induced vascular complications in both WT and SMC-IKK2–KO mice. Taken together, these data suggest that VSMC death is a critical step in the pathogenesis of CKD-dependent medial calcification. The mechanism by which deletion of the IKK2/NF-κB pathway induces apoptosis is the reduction in antiapoptotic genes. We have found in this study that the activation of NF-κB by the deletion of IκBα significantly induces 21 out of 22 antiapoptotic genes expressed in VSMCs.

As we mentioned, we are aware of several previous studies that proposed causative roles of the NF-κB pathway in CKD-induced vascular calcification (24–30). The direct conflicts between our current study and the previous studies could be due to the following reasons: (a) Indirect evidence — most of the previous studies concluded the contribution of the NF-κB pathway by indirectly showing changes in NF-κB signaling (25, 26). (b) Since some of the previous studies used RNAi, overexpression of IκBα, or dominant negative IκBα in vitro and in vivo, modulation of the NF-κB pathway was partial (<80%) and not tissue or cell specific. Instead, we completely and directly modulated the genes involved in the IKK2/NF-κB pathway by using CRISPR/Cas9 (100%) and SMMHC-Cre-*loxP* (>99%) techniques. (c) The study by Yoshida et al. used a Cre-*loxP* system to induce dominant negative IκBα in SMCs. However, the SM22-Cre line that they used induces Cre recombinase in SMCs, including myofibroblasts and macrophages (58). (d) Difference in animal models. Previous studies by Zhao et al. and Yoshida et al. (28, 30) used adenine-induced CKD rats and mice, respectively. We used 5/6 nephrectomy to induce CKD in mice. For a direct comparison, further studies are required to test whether SMC-IKK2 KO and SMC2-IκBα KO in mice affect vascular complications induced by adenine-induced CKD.

CKD induces chronic systemic inflammation and activates the NF-κB pathway in other tissues and cells such as macrophages, lymphocytes, and adipose tissues in addition to VSMCs (51, 59). We and other groups have previously shown that blocking specific proinflammatory cytokines such as TNF-α is effective in preventing vascular calcification in animal models (14, 15). We still believe that the inhibition of systemic inflammation is beneficial for blocking CKD-mediated medial calcification. Several previous studies have also shown that systemic inhibition of the IKK2/NF-κB pathway by chemical inhibitors or RNAi also blocks vascular calcification (28, 55). Monocytes could be a major source of increased proinflammatory cytokines in CKD, although unlike atherosclerotic CKD models, macrophage infiltrations in the aorta were not observed in our CKD mouse model of medial calcification. Further studies are required for identifying how tissue/cell inflammation and proinflammatory mediator production play a major role in regulating CKD-induced medial calcification.

In this study, we unexpectedly demonstrate that activation of the IKK2-mediated pathway works as a safeguard for VSMCs from ectopic mineralization in CKD by blocking cell death–mediated activation of calcifying–EV secretion. In addition, this study suggests that proinflammatory cytokines produced locally by VSMCs have more minor effects on the pathogenesis of CKD-mediated medial calcification than we previously anticipated.

## Methods

### Sex as a biological variable.

We used only males for this study because the SMMHC-CreER^(T2)^ transgene is located on the Y chromosome and male mice are more susceptible to CKD-dependent vascular complications, as we described previously (42, 53).

### Animals.

SMMHC-GFP (stock 007742, Jackson Laboratory), SMMHC-CreER^(T2)^ (stock 019079, Jackson Laboratory), and CKD mice were generated as previously described (39, 42). IKK2 conditional KO (*IKK2^fl/fl^*) mice were provided by Michael Karin at the University of California San Diego (60). To generate IκBα conditional KO mice, we flanked exons 2–5 of the *Nfkbia* (IκBα) gene with 2 *loxP* sites (Supplemental Figure 3). The targeting vector PG00171_Y_4_H09–*Nfkbia* was purchased from the MMRRC Repository. The targeting vector was linearized with AsiSi and introduced by electroporation into murine B6/129 hybrid EC7.1 ES cells. Karyotypically normal ES clones were microinjected into C57BL/6 blastocysts to produce chimeric founders at the Bioengineering Core Facility at the University of Colorado Anschutz Medical Campus. The *IκBα^fl/fl^* mice were crossed with Rosa-Flp mice to remove the LacZ-Neo cassette. All of the mouse strains were backcrossed at least 10 times with DBA/2J mice that are susceptible to CKD-dependent medial calcification. The DBA genetic background was checked by the PCR speed congenic service at the Molecular Biology Core Facility at the University of Colorado. To generate VSMC-specific IKK2-KO and IκBα-KO mice, *IKK2^fl/fl^* and *IκBα^fl/fl^* mice were intercrossed with SMMHC-CreER^(T2)^ mice to obtain SMMHC-CreER^(T2)^; *IKK2^fl/fl^* mice and SMMHC-CreER^(T2)^; *IκBα^fl/fl^* mice, respectively. SMMHC-CreER^(T2)^ mice were used as control mice. As shown in Supplemental Figure 4G, 8-week-old mice were subjected to 5/6 nephrectomy to induce CKD as previously reported, whereas sham operation was used as an NKD condition (38, 42, 53, 54, 61). One week after the surgeries, CKD mice were injected intraperitoneally with tamoxifen (40 mg/kg body weight, 5 consecutive days) in vegetable oil. After the injections, SMC-IKK2–KO and SMC-IκBα–KO mice were maintained on a special diet (TD110198, ENVIGO) for 3 and 12 weeks, respectively, unless indicated otherwise. The special diet contains by weight 19.5% casein, 0.3% D,L-methionine, 41.6% sucrose, 7.5% maltodextrin, 21% anhydrous milk fat, 0.15% cholesterol, 4.8% cellulose, 1.3 % Ca-P deficient mineral mixture, 1.9% calcium phosphate, 0.7% potassium phosphate, 0.2% calcium carbonate, 1.0% vitamin mixture, 0.03% ethoxyquin, and 0.02% pink food color. CKD SMC-IKK2–KO and WT mice were injected intraperitoneally daily with either GSK2656157 (Cayman Chemical, 17372; 0.5 mg/kg body weight) or vehicle (6% DMSO/PBS) for 3 and 12 weeks, respectively. Calcified lesions in the aortic arches were analyzed as previously described using von Kossa staining (38, 39, 42, 53, 61, 62). The calcified areas in 25 sections were determined in a blinded fashion by light microscopy. Cell death was detected using an In Situ Cell Death Detection Kit (Roche, 12156792910). CD63^+^ALP^+^ calcifying EVs in the aortic sinus were detected using Alexa Fluor 488–conjugated anti-CD63 polyclonal antibody (Novus, clone MX-49.129.5) and Alexa Fluor 568–conjugated anti-ALP recombinant antibody (SelleckChem, A5111), as previously described (39, 42, 54, 63). At least 5 sections and 100 DAPI^+^ nuclei from each sample were analyzed.

### aPWV.

aPWV was assessed noninvasively using an Indus Doppler Flow Velocity System (Scintica) as previously described (64, 65). Briefly, isoflurane (2%) was used to anesthetize mice that were placed supine with legs secured to ECG electrodes on a heated board. Doppler probes were placed on the skin at the transverse aortic arch and abdominal aorta approximately 4 cm apart. For each site, the pre-ejection time, or time between the R-wave of the ECG to the foot of the Doppler signal, was determined. To calculate aPWV, the distance between the probes was divided by the difference between the thoracic and abdominal pre-ejection times and is presented as centimeters per second (cm/s). Following aPWV measures, mice were euthanized by exsanguination via cardiac puncture while anesthetized with isoflurane.

### Cell cultures.

Human primary VSMCs and mouse immortalized VSMCs were purchased from Applied Biological Materials (MOVAS1) and American Type Culture Collection (CRL-2797), respectively. VSMCs were maintained in DMEM containing 1% exosome-depleted FBS (A2720801, Thermo Fisher Scientific) with 100 U/mL penicillin and 100 μg/mL streptomycin. VSMCs were treated with either 1 ng/mL human TNF-α (GenScript, Z00100) with 2.4 mM inorganic phosphate or 2.4 mM inorganic phosphate alone in the absence or presence of 0.1 μM GSK2656157.

### CRISPR/Cas9 system–based genetic KO of IKK2, IκBα, and NFKB1 genes.

*IKK2*, *IκBα*, and *NFKB1* gene sgRNAs were cloned into the LentiCRISPRv2 plasmid (Addgene, 98290) as previously described (54, 63). The sgRNA sequences are shown in Supplemental Table 5. HEK293T cells were seeded at 6 × 10^5^ cells/well in 6-well plates, grown overnight, and then transfected with 300 ng psPAX2, 100 ng pMD2, and 400 ng of each sgRNA CRISPR/Cas9 lentiviral plasmid (plasmid amount ratio 3:1:4) using Turbofect transfection reagent (Thermo Fisher Scientific). Lentiviral media were centrifuged once at 1500*g* for 3 minutes and the supernatant was collected. VSMCs were seeded in 6-well plates and infected 24 hours later with each lentiviral medium in the presence of 10 μg/mL polybrene. Cells were treated with 5 μg/mL puromycin for selection of infected cells. Total RNA of heterogeneous cells was collected and cDNA synthesis was conducted from the RNA template, followed by high-resolution melting analysis with a StepOne Plus qPCR instrument (Applied Biosystems) to check for mutations occurring in regions around *IKK2*, *RelA*, and *NFKB1* sgRNA target sequences. Heterogeneous cells with gene mutations were plated at 0.5 cells/well in a 96-well plate to obtain a single-cell clone. Protein from gene-edited clones was prepared and analyzed by immunoblotting to determine whether gene KO was complete.

### Generation of VSMCs expressing IKK2DN.

FLAG–*IKK2DN* (Addgene, 15466) was cloned into the pLenti-CMV-Puro DEST vector (Addgene, 17452) using a Gateway cloning system (Invitrogen). VSMCs were infected with lentivirus containing FLAG-*IKK2DN*. Cells were treated with 5 μg/mL puromycin for selection of infected cells. The single clones were analyzed by immunoblotting with an anti-FLAG antibody (clone M2, Sigma-Aldrich).

### RNA analysis.

Total RNA was isolated using a Direct-zol RNA kit (Zymo Research). cDNA was synthesized from 500 ng total RNA using a High-Capacity cDNA Reverse Transcription Kit (Applied Biological Materials Inc). qRT-PCR was performed using an Applied Biosystems StepOne Plus qPCR instrument with SYBR Select Master Mix according to the manufacturer’s instructions. Primer sequences are shown in Supplemental Table 5.

### Calcium content in cultured cells and aortas.

For evaluation of vascular mineralization, VSMCs were plated at 1.0 × 10^5^ cells/well in a 12-well plate and grown overnight. VSMCs were treated with 1 ng/mL TNF-α or 2.4 mM P_i_ every 2 days for 6 days. VSMCs were incubated with 0.6N HCl overnight at 4°C. After incubation, 0.6N HCl was collected to measure calcium content, and then VSMCs were lysed with 0.1N NaOH/0.1% SDS to measure protein concentration with a BCA assay. Aortas were collected from mice and stored at –20°C. Dried aortas were defatted with chloroform and methanol (2:1) for 48 hours and dehydrated with acetone for 3 hours. The dried samples were incinerated to ashes at 600°C for 24 hours using an electric muffle furnace (Thermo FIsher Scientific), and then extracted with 0.6N HCl. Calcium content from cultured cells and aortas was quantified using the *o*-cresolphthalein method. In addition, VSMCs were stained with alizarin red to identify calcium deposits 6 days after TNF-α and P_i_ treatments (42, 53, 54).

### RNA-seq.

Total RNA was isolated using a Direct-zol kit. mRNA-seq library construction and sequencing was performed at BGI America (http://www.bgi.com) in accordance with the manufacturer’s instructions using a DNBseq system as previously described (62, 63).

### Cell sorting.

To isolate aortic VSMCs, isolated aortas were digested to single cells by digestion at 37°C in collagenase buffer (3.2 mg/mL collagenase II, 0.7 mg/mL elastase, 0.2 mg/mL soybean trypsin inhibitor) in Hank’s buffered saline solution as previously described (42, 53). Aortas were harvested under sterile conditions following flushing of the vasculature system with sterile heparinized PBS and minced prior to digestion. Single-cell suspensions were sorted based on GFP for SMMHC-GFP mice under sham operation and CKD. Sorting was performed on a MoFlo high-speed cell sorter at the University of Colorado flow cytometry and sorting core facility.

### p65 translocation assay.

WT and IKK2-KO VSMCs were seeded onto 35-mm glass-bottom imaging dishes (no. 1.5 cover glass). When cells reached approximately 70%–75% confluence, 2.5 μg of *EGFP-p65* (Addgene, 111190) was transfected using Lipofectamine 2000 (Thermo Fisher Scientific). Twenty-four hours after transfection, cells were imaged in imaging buffer supplemented with 0.1% BSA and imaged with a Zeiss LSM 780 microscope with a C-Apochrome 40×/1.20 W Korr FCS M27 objective using the 488 nm wavelength laser in the presence of 1 μg/mL TNF-α at 1 frame every 5 seconds for 1 hour. Regions of interest were drawn over the nucleus and cytosol of cells and the nuclear fluorescent signal was divided by the cytosolic fluorescent signal to obtain the fluorescence ratio.

### EMSA.

EMSA analysis was performed as previously described (66, 67). The DNA-binding activity of NF-κB was assayed according to the protocol from Promega Corp. Briefly, the oligonucleotide with NF-κB consensus binding element (Promega) was end labeled by T4 polynucleotide kinase (Promega) using [γP^32^]-ATP (Bio-Rad). Total protein extract (30 μg) was isolated from the VSMCs using 1× Passive Lysis Buffer (Promega) and was mixed with radiolabeled oligonucleotide for binding. Unlabeled cold probe was used to compete with the radiolabeled probe to show binding specificity. The reaction mixture was loaded in a 5% polyacrylamide gel under nondenaturing conditions and resolved by electrophoresis at 4°C. The gel was then dried and exposed to x-ray film to visualize the binding of NF-κB to the radiolabeled probe. The binding specificity was previously shown by blockage of binding with excess competitive cold probe, and the position of the NF-κB p65-p50 complex was confirmed using anti-p65 (Cell Signaling Technology, catalog 8242) and anti-p50 antibodies (Thermo Fisher Scientific, catalog 14-6732-81) (66–68).

### Immunoblot analysis.

Cell and tissue lysates were prepared using RIPA buffer (150 mM NaCl, 1% Nonidet P-40, 0.5% sodium deoxycholate, 0.1% SDS, 50 mM Tris, pH 8.0). Cells were disrupted by pipetting 10–15 times, centrifuged at 13,800*g* for 10 minutes at 4°C, and the supernatant was collected for total cell lysates. The samples were separated by SDS-PAGE, transferred to a nitrocellulose membrane, and immunoblotted with antibodies against the following proteins: IKK2 (clone D30C6, catalog 8943), p-IKK2 (clone 16A6, catalog 2697), IκBα (clone 44D4, catalog 4812), p-IκBα (clone 14D4, catalog 2859), p65 (clone D14E12, catalog 8242), p-p65 (clone 93H1, catalog 3033), p50 (clone D4P4D, catalog 13586), annexin A2 (clone D11G2, catalog 8235), p-MLKL (clone D6E3G, catalog 37333), MLKL (clone D6W1K, catalog 37705), and Casp3 (catalog 9662) from Cell Signaling Technology; GAPDH (0411) (catalog sc-47724) from Santa Cruz Biotechnology; and β-actin (catalog 66009) from Proteintech. Annexin 6 (catalog A305-309A), CD63 (catalog 143901, clone NVG-2), and TNAP (clone A511) antibodies were purchased from Bethyl, BioLegend, and Selleck, respectively. Samples were visualized using horseradish peroxidase coupled to appropriate secondary antibodies with enhancement by an ECL detection kit (Thermo Fisher Scientific).

### Immunohistochemistry.

Calcified aortas from a patient (African American male, 86 years old) with CKD were obtained from the Cooperative Human Tissue Network. The frozen serial sections were stained with von Kossa or anti–p-p65 antibody (clone 93H1, Cell Signaling Technology, catalog 3033).

### NTA.

NTA was performed at the University of North Carolina Nanomedicines Characterization Core Facility in accordance with the manufacturer’s instructions using a NanoSight NS500 (Malvern Panalytical).

### Isolation of ApoBDs and EV fraction.

A 200 mL cell culture medium was centrifuged at 300*g* for 10 minutes twice. The collected supernatant was centrifuged for 20 minutes at 3,000*g* twice. The pellet was resuspended in 1× PBS as the ApoBD-enriched fraction. The 3,000*g* supernatant was centrifuged for 20 minutes at 15,000*g* twice. The collected supernatant was further ultracentrifuged for 1 hour at 100,000*g*. The 100,000*g* pellet was washed with 1× PBS and re-centrifuged for 1 hour at 100,000*g* as the EV-enriched fraction (48, 69, 70).

### Statistics.

Data were collected from more than 2 independent experiments and are reported as mean ± SEM. Statistical analysis for 2-group comparison was performed using the Student’s *t* test, or 1-way ANOVA or 2-way ANOVA with a Newman-Keuls post hoc test for multiple group comparisons. Significance was accepted at *P* less than 0.05.

### Study approval.

All animal protocols and experimental procedures were approved by the Institutional Animal Care and Use Committee at the University of Colorado Anschutz Medical Campus.

### Data availability.

All data included in all figures are fully available in Excel format in the Supporting Data Values file included in the supplemental material. The mRNA-seq raw data were deposited to the NCBI Gene Expression Omnibus (GEO GSE229679).

## Author contributions

M Masuda, SMA, JGM, YS, and M Miyazaki developed the methodology. M Masuda, SMA, JGM, and M Miyazaki analyzed data. M Masuda, JGM, SMA, and M Miyazaki performed the experiments and collected data. M Miyazaki wrote the original draft of the manuscript, which was reviewed and edited by ALK, YS, M Masuda, and M Miyazaki. M Masuda, SMA, JGM, and M Miyazaki generated figures. M Miyazaki conceptualized and supervised the study and acquired funding.

## Supplementary Material

Supplemental data

Unedited blot and gel images

Supplemental tables 1-5

Supplemental video 1

Supplemental video 2

Supporting data values

## Version 1 03/12/2024

In-Press Preview

## Version 2 04/08/2024

Electronic publication
