# Supplementary material for: Activation of the IKK2/NF-κB pathway in VSMCs inhibits calcified vascular stiffness in CKD
Source: JCI Insight. 2024 Mar 12;9(7):e174977. doi: 10.1172/jci.insight.174977 (PMC11128211; doi:10.1172/jci.insight.174977)
Supplement: Supplemental data [file jciinsight-9-174977-s142.pdf]

Supplemental Video legends

Supplemental Video 1. Time lapse imaging of EGFP-p65 translocation in wild-type VSMCs expressing GFP-p65. Images of wild-type VSMCs expressing GFP-p65 treated with TNFα were acquired for one hour. Related to Figure 2B.

Supplemental Video 2. Time lapse imaging of EGFP-p65 translocation in IKK2KO VSMCs expressing GFP-p65. Images of IKK2KO VSMCs expressing GFP-p65 treated with TNFα were acquired for one hour. Related to Figure 2B.
