# Supplementary material for: Activation of the IKK2/NF-κB pathway in VSMCs inhibits calcified vascular stiffness in CKD
Source: JCI Insight. 2024 Mar 12;9(7):e174977. doi: 10.1172/jci.insight.174977 (PMC11128211; doi:10.1172/jci.insight.174977)
Supplement: Unedited blot and gel images [file jciinsight-9-174977-s143.pdf]

Fig 1C

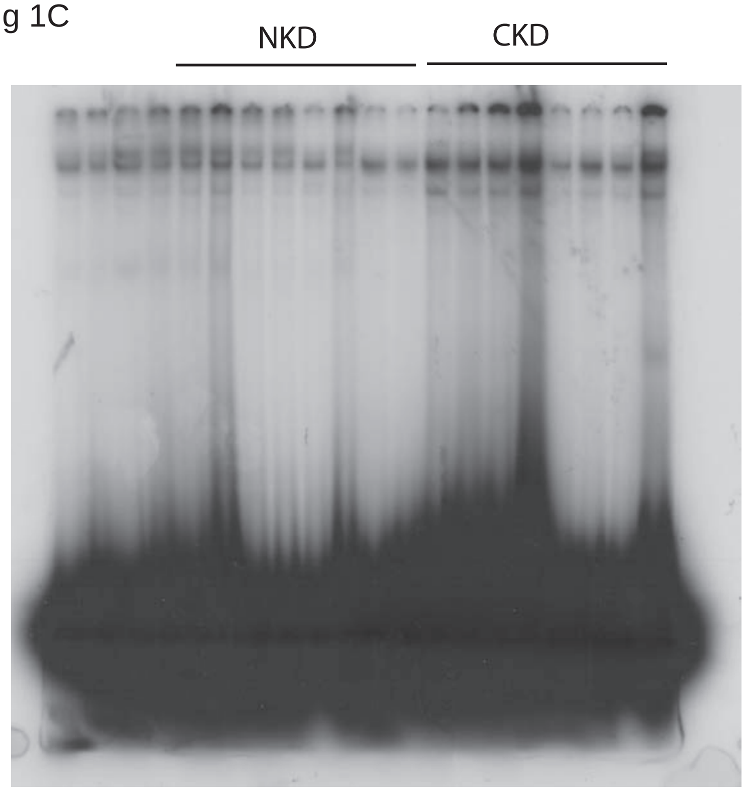

Fig 1D

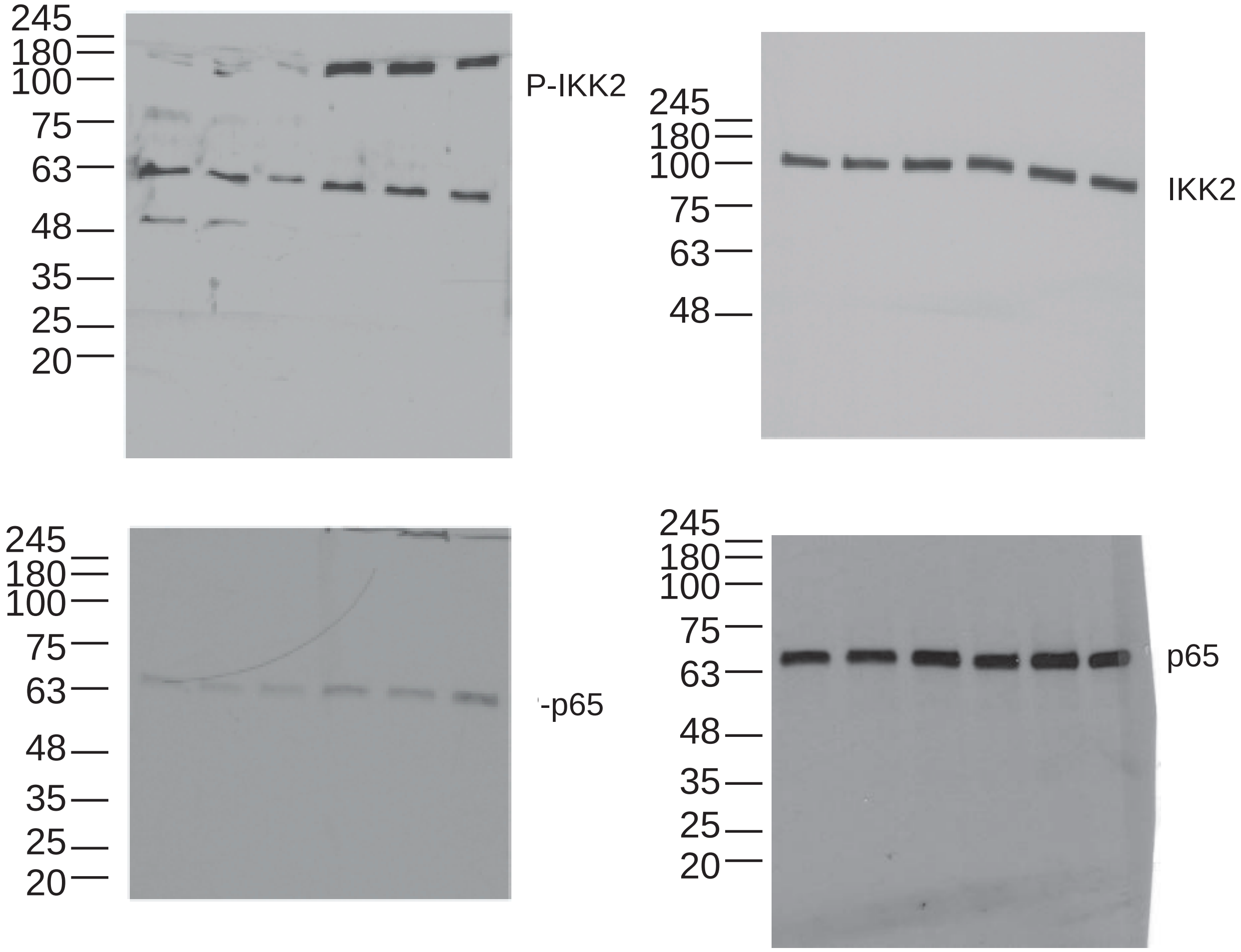

Figure 1

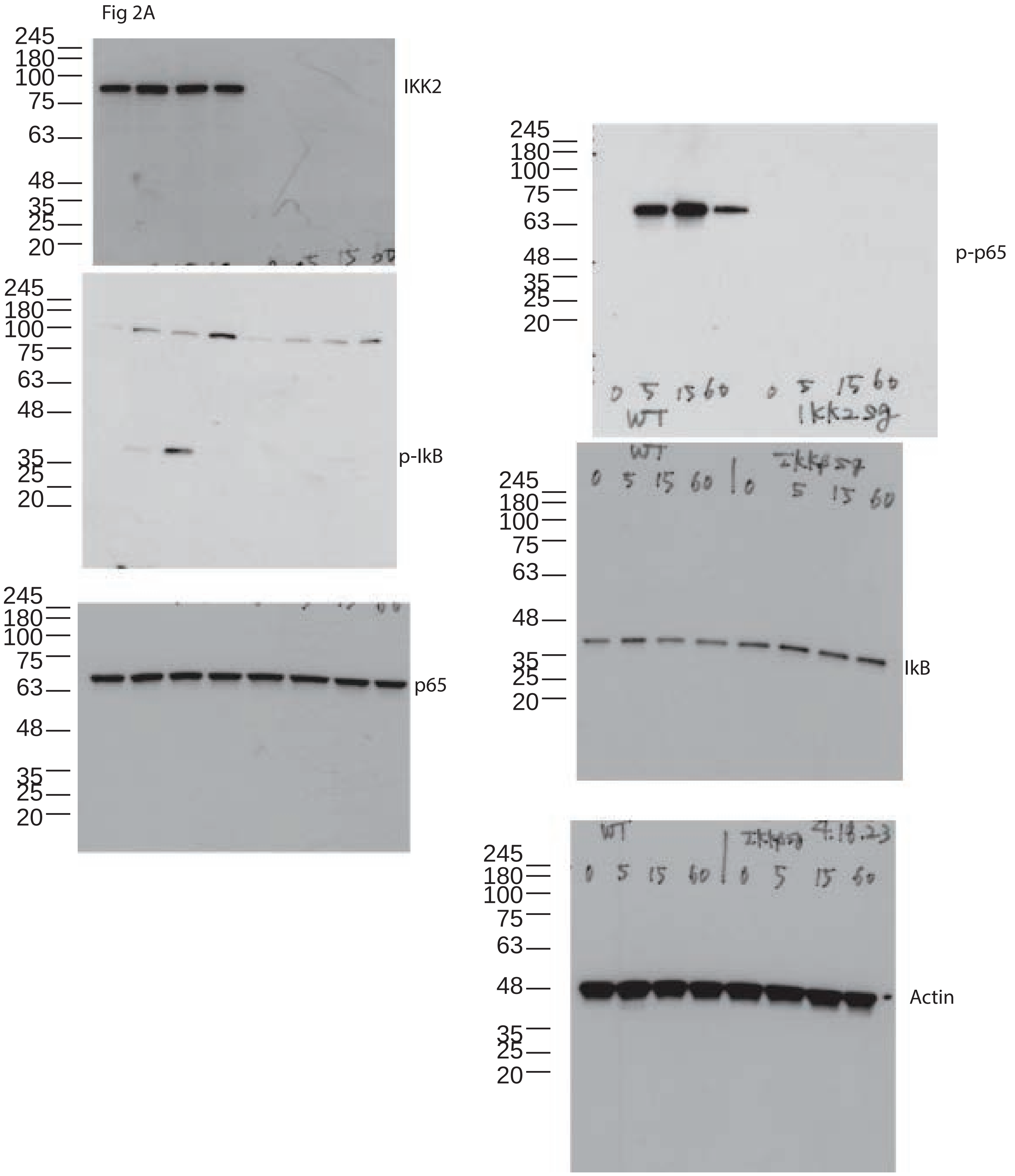
Fig 2A
245
180
100
75
63
48
35
25
20
IKK2
p-IkB
p65
p-p65
IkB
Actin
0 5 15 60
WT

Figure 2

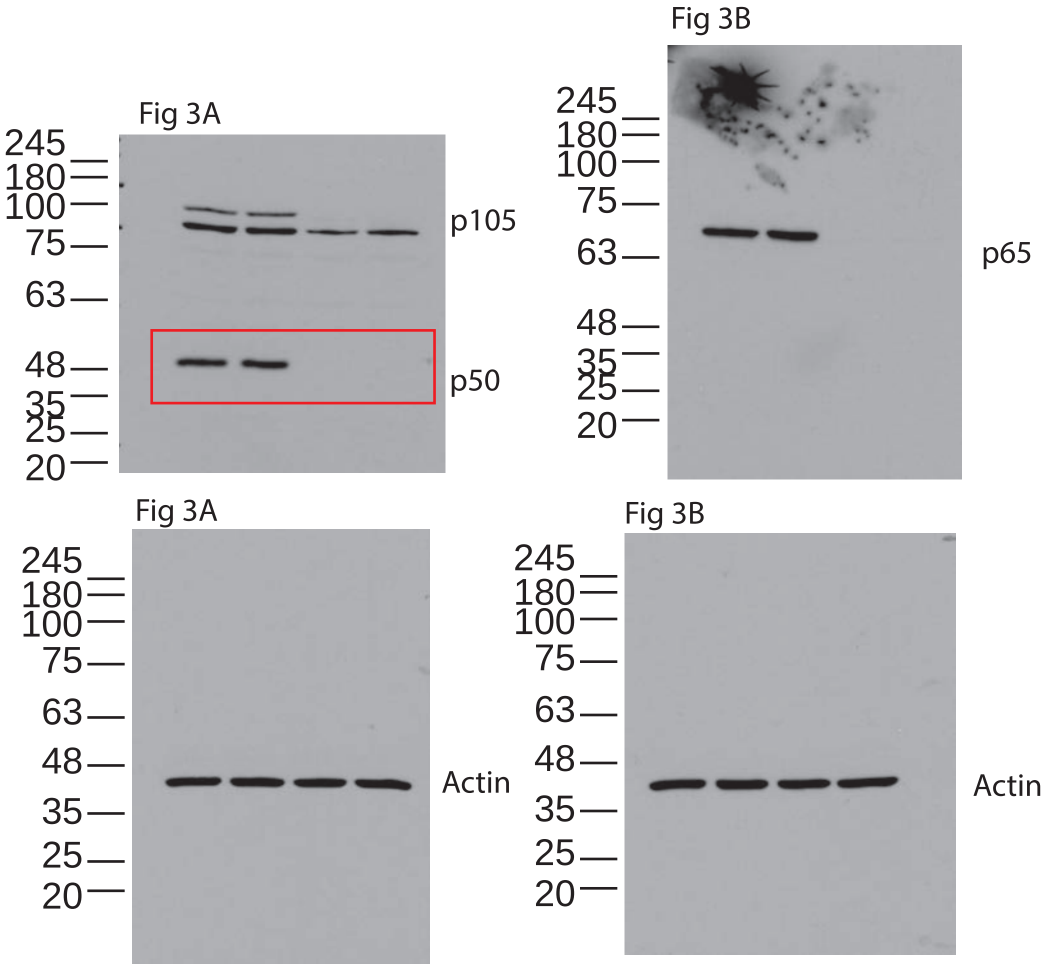
Fig 3A
245
180
100
75
63
48
35
25
20
p105
p50
Fig 3B
245
180
100
75
63
48
35
25
20
p65
Fig 3A
245
180
100
75
63
48
35
25
20
Actin
Fig 3B
245
180
100
75
63
48
35
25
20
Actin

Figure 3

Fig 4A

Figure 4

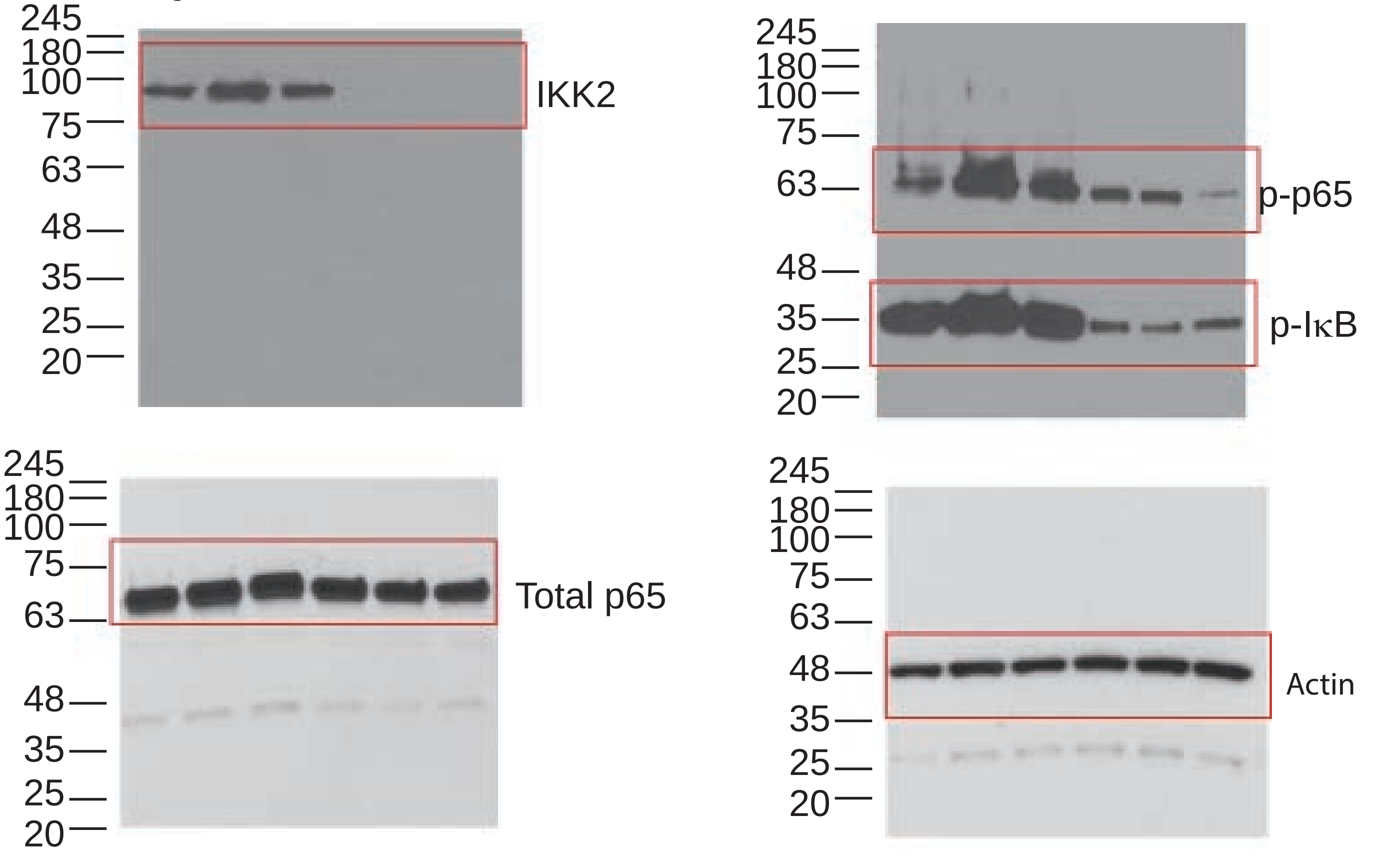

Fig 5A

Figure 5

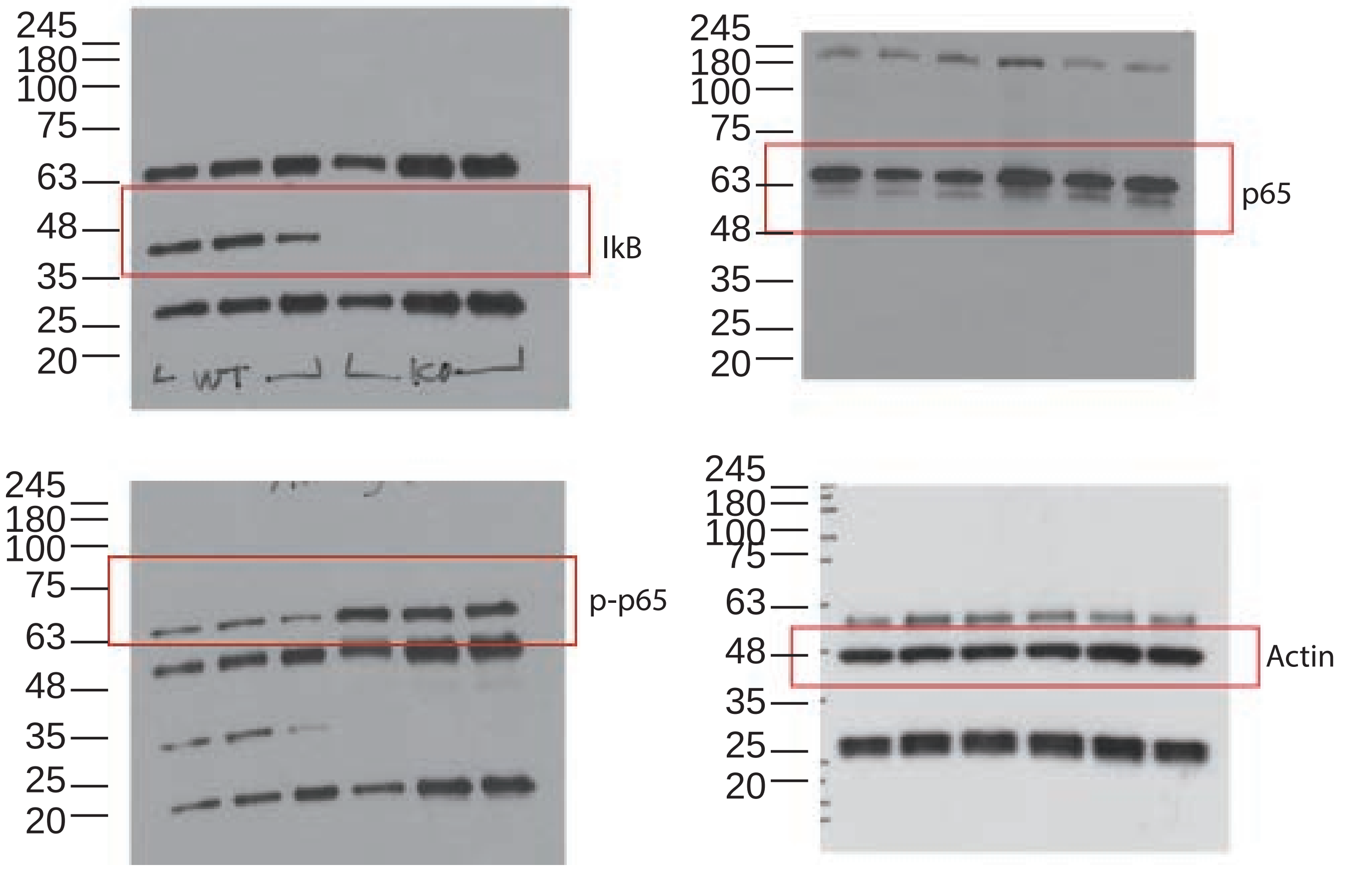

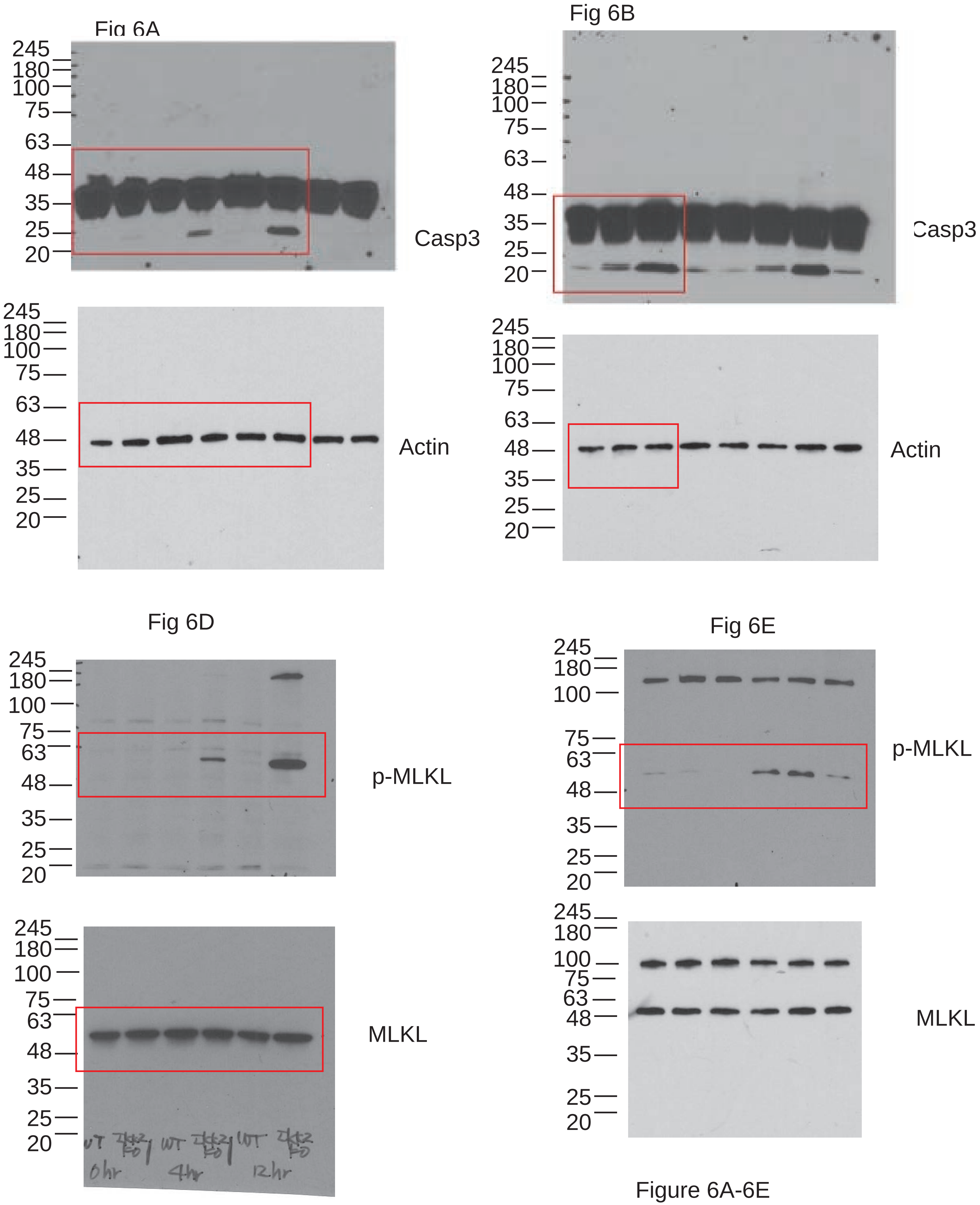
Fig 6A
245
180
100
75
63
48
35
25
20
Casp3
Actin
Fig 6B
Casp3
Actin
Fig 6D
p-MLKL
MLKL
WT
WT
WT
0hr
4hr
12hr
Fig 6E
p-MLKL
MLKL

Figure 6A-6E

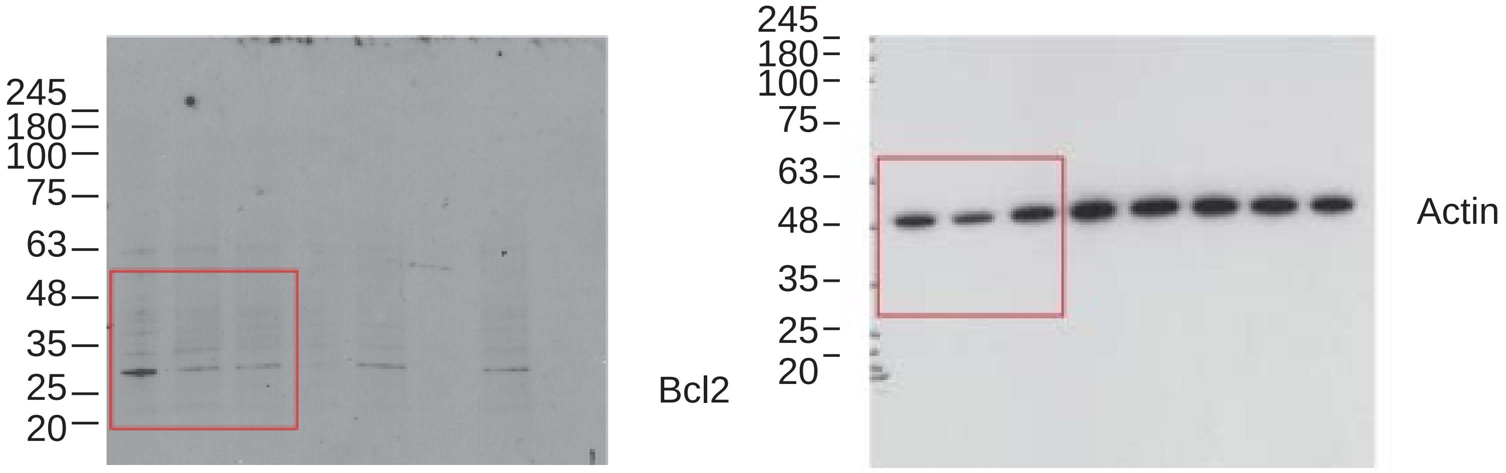

Figure 6F

Fig 7F

Fig 7J

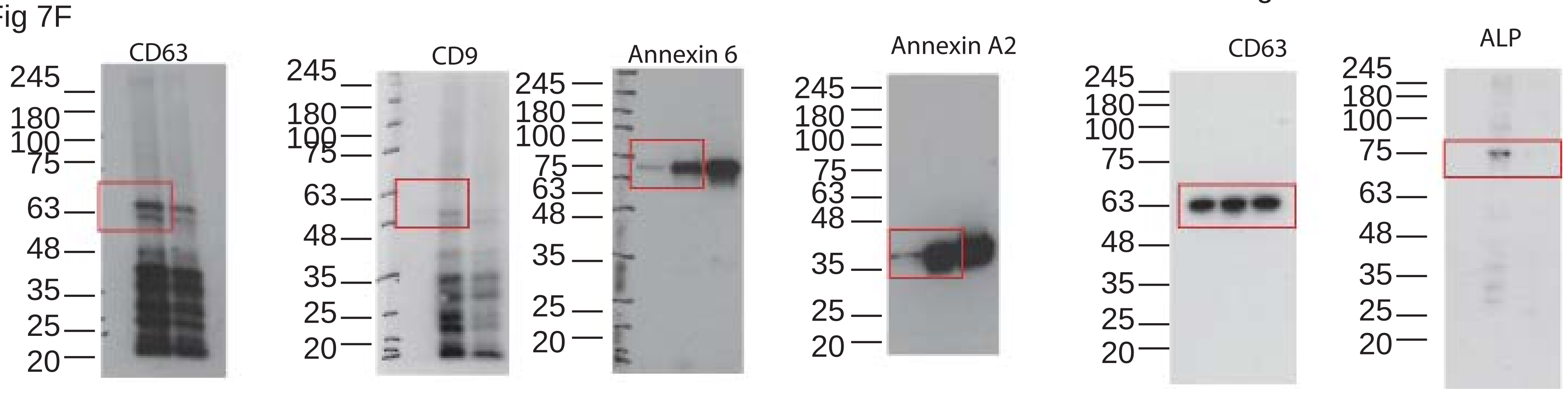

Figure 7

S. Fig 2

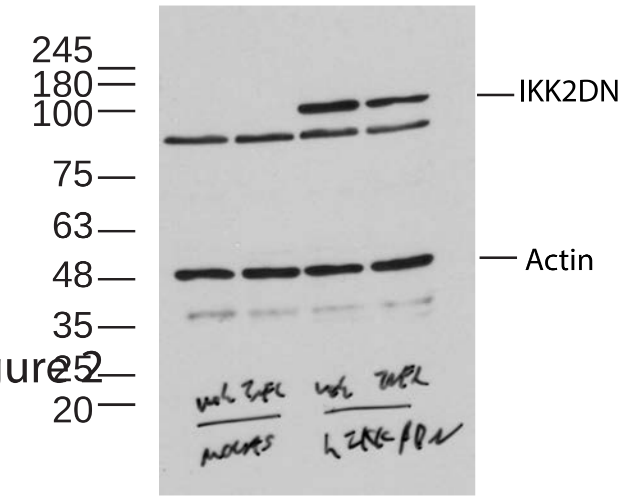

Figure2

Suppl Figure 2

S. Fig 3A

S. Fig 3D

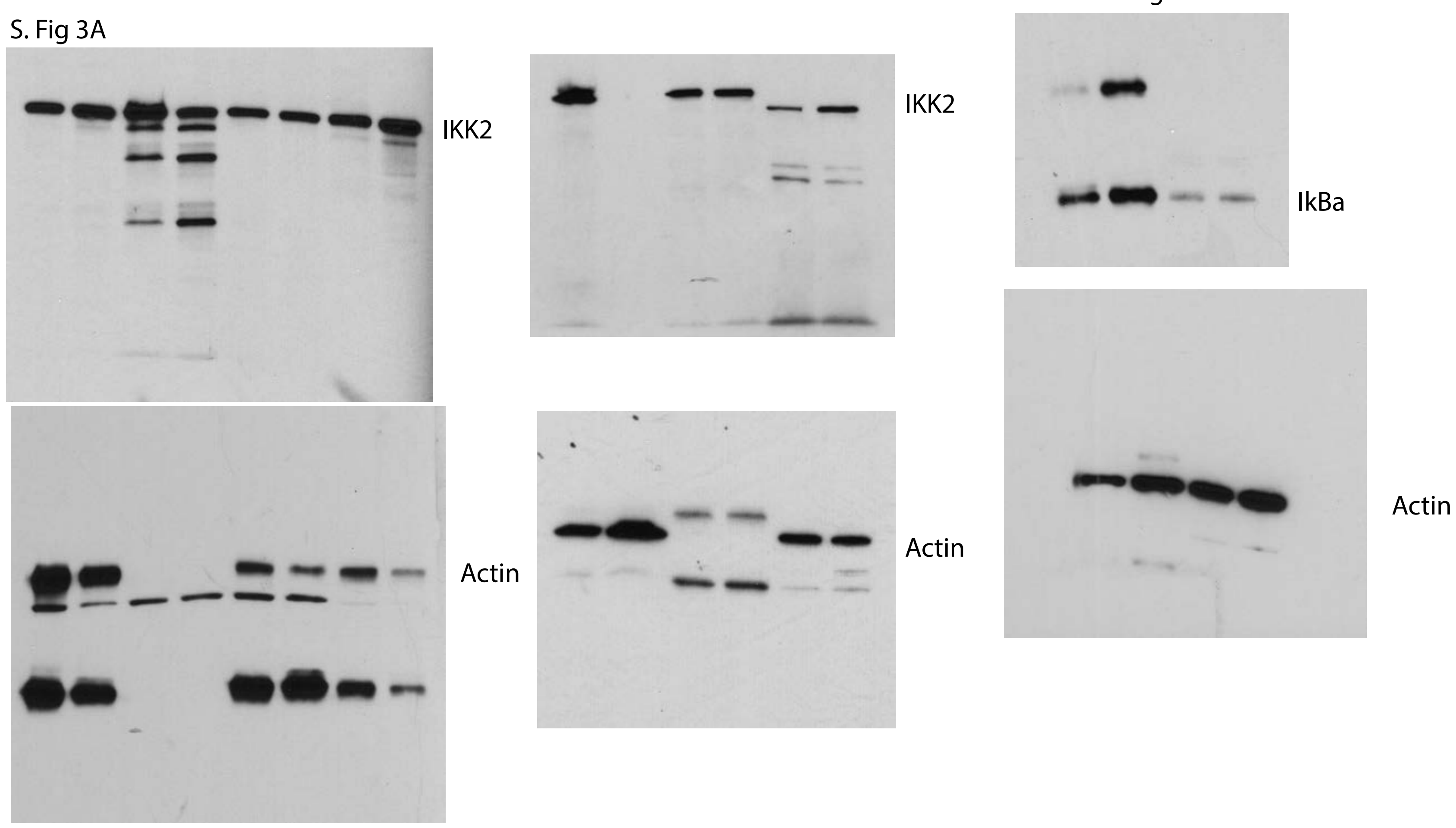

Suppl Figure 3
